# Transcriptomics Analysis Reveals a Putative Role for Hormone Signaling and MADS-Box Genes in Mature Chestnut Shoots Rooting Recalcitrance

**DOI:** 10.3390/plants11243486

**Published:** 2022-12-13

**Authors:** Jesús Mª Vielba, Saleta Rico, Nevzat Sevgin, Ricardo Castro-Camba, Purificación Covelo, Nieves Vidal, Conchi Sánchez

**Affiliations:** 1 Misión Biológica de Galicia, Consejo Superior de Investigaciones Científicas, 15780 Santiago de Compostela, Spain; 2 Department of Horticulture, University of Sirnak, 73100 Sirnak, Turkey

**Keywords:** adventitious rooting, auxin, chestnut, mads-box, maturation, phytohormones, recalcitrance, transcriptomics

## Abstract

Maturation imposes several changes in plants, which are particularly drastic in the case of trees. In recalcitrant woody species, such as chestnut (*Castanea sativa* Mill.), one of the major maturation-related shifts is the loss of the ability to form adventitious roots in response to auxin treatment as the plant ages. To analyze the molecular mechanisms underlying this phenomenon, an in vitro model system of two different lines of microshoots derived from the same field-grown tree was established. While juvenile-like shoots root readily when treated with exogenous auxin, microshoots established from the crown of the tree rarely form roots. In the present study, a transcriptomic analysis was developed to compare the gene expression patterns in both types of shoots 24 h after hormone and wounding treatment, matching the induction phase of the process. Our results support the hypothesis that the inability of adult chestnut tissues to respond to the inductive treatment relies in a deep change of gene expression imposed by maturation that results in a significant transcriptome modification. Differences in phytohormone signaling seem to be the main cause for the recalcitrant behavior of mature shoots, with abscisic acid and ethylene negatively influencing the rooting ability of the chestnut plants. We have identified a set of related MADS-box genes whose expression is modified but not suppressed by the inductive treatment in mature shoots, suggesting a putative link of their activity with the rooting-recalcitrant behavior of this material. Overall, distinct maturation-derived auxin sensibility and homeostasis, and the related modifications in the balance with other phytohormones, seem to govern the outcome of the process in each type of shoots.

## 1. Introduction

Adventitious rooting (AR) is a highly complex developmental process in which roots regenerate from tissues other than the primary root, mainly stems and leaves, as a plant response to overcome stressful situations or traumatic damages [1]. De novo root regeneration is usually initiated from cells that have not been developmentally specified to form roots [2]. The successful development of adventitious roots (ARs) requires the proper integration of multiple internal and external cues. These signals include physiological status and age of the plant, genotype, carbohydrate and nutrient availability, light conditions or biotic factors that might exert a significant influence on several molecular facets such as microtubule and cell wall dynamics, cell cycle, transcriptional activity, epigenetics modulations or microRNAs [3]. Phytohormones emerge as the key factors in the integration and processing of all the information, with their dynamic and fast response as well as the balance between different families outlining the outcome of the process.

The formation of ARs is commonly divided in three sequential steps, historically defined according to the histological detected events, and their identity has been consolidated by molecular findings [4]. During the induction phase, certain cells in the vasculature or neighboring zones respond to the inductive stimuli and modify their developmental fate, changing their ongoing genetic program and becoming root founder cells (RFCs). Deep modification of the gene expression pattern in RFCs results in an asymmetric cell division that establishes the polarity of the new organ, ending the induction stage and giving way to the initiation phase, in which the developing root grows through the tissues of the plant, and acquires meristematic identity and self-organizing ability. Eventually, the new root emerges across the epidermis during the expression phase and vascular connections with the stem base are established [5]. The induction phase appears to be the tipping point of the process, because once the identity of the RFCs is established, the development of the new organ proceeds through controlled cell divisions of meristematic cells and specific modifications of the surrounding tissues without any further cell reprogramming [6]. However, in many species, an early phase taking place before or at the beginning of the induction phase is required. During this step, called dedifferentiation, cells need to erase their developmental program and return to a less differentiated state before being able to respond to the inductive stimuli. This phase demands a profound genetic reprogramming that results in the enhanced developmental plasticity of the cell [7]. This loss of the previously acquired fate of the cells is a central idea in plant regeneration studies [2].

Vegetative propagation of forest and ornamental species relies on the correct formation of ARs in cuttings, which are induced by two main stimuli, wounding and auxin. First, wounding triggers changes in the hormone content and sensibility in the tissues, leading to the local accumulation of auxin in the wounding zone via activity of the auxin transport proteins [8]. However, in many species this initial endogenous supply is not enough for the induction of ARs, and exogenous auxin, mainly in the form of indol-3-butyric acid (IBA), is applied to the cut surface to ensure adequate content of this hormone, which acts as the master regulator of the induction phase [9,10]. Auxin is then asymmetrically distributed within the plant tissues in response to the activity of the auxin transport machinery, leading to the generation of a hormone gradient. Those cells in which auxin accumulate are tagged as the RFCs [11] and govern the subsequent steps of AR. Noteworthy, species- and genotype-dependent effects might refine different aspects of this process.

Nonetheless, the scenario is far more complex, as many other factors influence auxin homeostasis at the wounding/rooting zone. Auxin signaling is a clear and well-defined pathway; however, its components might be controlled and modified by several other agents such as phytohormones and environmental cues. This sophisticated mechanism results in a wider range of auxin sensibility levels and broadens the repertoire of possible outcomes [12]. Hence, the modifications in the specific balance between auxin and other phytohormones adjust plant responses accordingly. Wounding is followed by an initial burst of jasmonic acid (JA) and ethylene (ET) in cuttings of several species [9,13], which is a common response to this type of stress in plants [14]. Both phytohormones seem to be crucial for AR, at least during the induction phase, while gibberellins (GAs), abscisic acid (ABA) and cytokinins (CKs) might exert a negative influence [15]. Nonetheless, the knowledge gathered is still fragmented and several questions remain unanswered, such as the transferability of results between species or the identity of relevant genes involved in the process. The formation of ARs in cuttings of many species is hampered by the recalcitrant character of the tissues to root regeneration in response to the applied stimuli, a behavior that is intensified with the aging of the plants, as mature tissues show an increased inability to form roots in response to inductive treatments [2]. Noteworthy, the induction of ARs relies on the auxin gradient rather than on the hormone concentration per se. In forest species, it has been shown that the inability of auxin-treated mature cuttings to create such hormone gradients derives in the formation of xylem tissue or callus structures [16,17,18,19]. Chestnut (*Castanea sativa* Mill.) is an appreciated and highly valuable tree widespread in the Mediterranean basin, with significant ecological and economic impacts. As with other species from the Fagaceae family, it presents a recalcitrant behavior that is more accentuated in mature tissues, thus limiting vegetative propagation and biotechnological approaches to increase its profitability. Knowledge concerning AR in chestnut species has recently been reviewed [7]; however, information is still scarce at present and the molecular basis of the AR process or recalcitrant behavior remains largely unknown. An in vitro working system comprising two chestnut lines derived from basal sprouts (BS) growing at the base of the trunk and from crown (CR) branches of the same field-grown tree was established in our lab. While BS shoots present a juvenile-like character and root easily when treated with exogenous auxin, mature shoots of the CR line do not root (reviewed in [7]). The system enables for the analysis of maturation-related responses at the phenotypical, biochemical and molecular levels.

Using long read sequencing technology, an in-house transcriptomics analysis was developed to study the differential genetic responses of juvenile-like and mature tissues to the root induction treatment (wounding and exogenous auxin). Given the genotype identity between both lines and their distinct behavior in response to the treatment, the detected Differentially Expressed Genes (DEGs) might be directly related to AR or to recalcitrance. Gene ontology and KEGG analysis helped to elucidate the molecular basis of the differential response to auxin and wounding in both types of tissues, and the potential role played by different phytohormones in this context. Particularly, a set of MADS-box genes was found to be highly expressed in mature shoots while they were barely detected in juvenile shoots. Expression characterization of some of these genes during the first hours after the inductive treatment suggests a close link between MADS-box genes’ activity and the recalcitrant behavior of mature shoots.

## 2. Results

**Figure 1:**
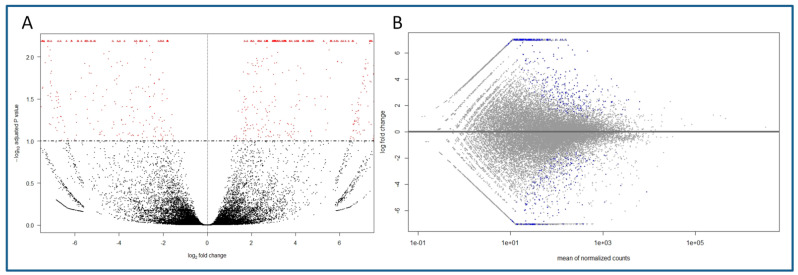
Overall results of the gene expression in the transcriptomics analysis of the BS and CR libraries, and identification of differentially expressed genes (DEGs). (**A**) Volcano plot showing the DEGs in CR samples (right, log2FC > 0) and BS samples (left, log2FC < 0) and the adjusted *p* value (<0.1). Red dots indicate DEGs for one condition or the other. (**B**) MA plot of the log2FC of the different genes versus the mean of normalized counts. Blue dots indicate DEGs (adjusted *p* value < 0.1).

The nanopore transcriptomics analysis generated 9,580,836 reads for the six libraries (BS01, BS02, BS03, CR01, CR02, CR03) together, totaling 5.8 Gbases. The average quality score (Q) was 9.85, with no reads below Q < 7.0. The mean sequence length was 605 bp. Once the adapters were removed and the quality of reads was checked, the obtained FastQ files were mapped against the *Q. suber* genome. For the three juvenile-like libraries, 79.16% of the files were successfully mapped, while in the case of the CR libraries the percentage was 75.22%. Volcano and MA plots showing the overall results of the transcriptomics analysis are shown in Figure 1. To confirm the mapping of the reads against the reference genome, a visual inspection was carried out with the IGV software. As seen in the examples shown in Figure S1, the sequences mapped to the different exons in the genes, with low or no mapping to the intron or intergenic regions.

**Figure 2:**
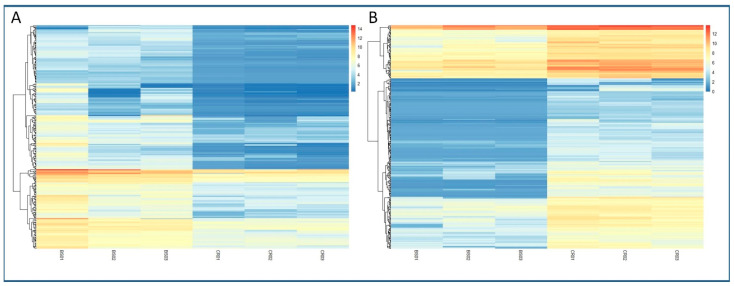
Heatmaps of the expression of DEGs detected in the differential analysis. Level of expression is shown as the log2FC of the transformed count data from the transcriptomics analysis. Different columns in the figure represent different samples, and different rows represent different genes. The colors from blue to red indicate increasing levels of expression. (**A**) Expression of DEGs detected in the BS samples. (**B**) Expression of DEGs detected in the CR samples.

The analysis allowed the identification of 392 DEGs in the CR samples (CR_DE), while 293 DEGs were identified in BS samples with respect to mature tissues (BS_DE). The complete list of DEGs in both types of tissues can be found in Tables S1 and S2. A heatmap showing the log2 Fold Change in expression of the DEGs for both conditions is shown in Figure 2. Within these DEGs, we detected a significant amount of uncharacterized genes, long non-coding RNAS (lncRNAs) and pseudogenes that were differentially expressed under one condition or the other (Table S3). In addition, a relevant number of TFs were also detected in the BS_DE and CR_DE libraries (Tables S4 and S5). Three out of the fourteen TFs identified in BS_DE belong to the GRAS family. Other relevant TFs were *LOB21*, *ATHB-14* and the ethylene-responsive *RAP2-7* (see Table S4 for gene symbols). For the CR_DE library, nine members of the MADS-box TF family, seven from the *JOINTLESS-like* group and two from the related *Short Vegetative Phase* (SVP) group were found to be more expressed in mature tissues. Moreover, three ethylene-responsive TFs (*ERF98*, *ERF107* and *RAP2-3*), three NAC TFs (*NAC22*, *NAC104*, *NTM-1 like 9*) and two *MYB* TFs (*MYB41*, *MYB8*) were identified for being more expressed in mature tissues. 

**Figure 3:**
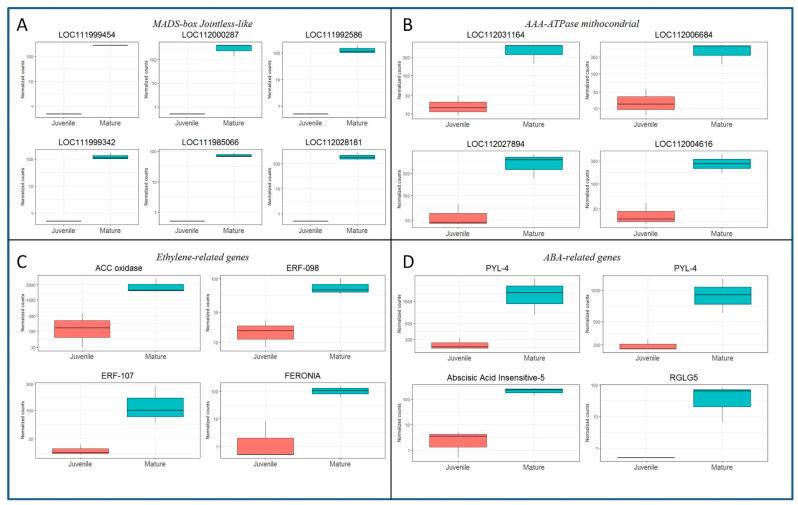
Boxplots showing the expression in normalized counts of DEGs detected in the CR samples versus their expression in BS samples. (**A**) Expression of six *MADS-box Jointless-like* genes. Gene symbols are given in the figure. (**B**) Expression of four *AAA ATPase mitochondrial* genes. Gene symbols are given in the figure. (**C**) Expression of ethylene-related genes: *ACC oxidase*, LOC111997351; *ERF-098*, LOC112018069; *ERF-107*, LOC112033868; *FERONIA*, LOC112001600. (**D**) Expression of Abscisic acid-related genes: *PYL-4*, LOC111988217 and LOC112017749; *ABSCISIC ACID-INSENSITIVE 5 protein 3* (*ABI-3*), LOC112026097; *RGLG-5*, LOC112025452.

The identified DEGs could be related to relevant signaling or molecular routes, according to their homologies. Boxplot graphs of the normalized counts were developed for several of the identified DEGs. The expression of six of the *MADS-box Jointless-like* genes detected in the CR_DE dataset are shown in Figure 3A. All of them exhibited similar expression level, suggesting that they might be co-regulated. Similarly, nine genes annotated as *AAA-ATPases*, from which seven are specifically annotated as *ASD mitochondrial-like*, were also preferentially expressed in CR shoots. The significant differences in expression between juvenile-like and mature shoots of four of those genes are shown in Figure 3B. Relevant genes involved in ET and ABA signaling were highly expressed in mature tissues as well. A *1-aminocyclopropane-1-carboxylate oxidase* (*ACC oxidase*) homolog (LOC111997351) was highly expressed in CR shoots (Figure 3C). This gene is a key factor in the biosynthesis pathway of ethylene, as it catalyzes the formation of ET from its precursor ACC. Moreover, other genes related to ET signaling, such as the TFs *ERF-098* (LOC112018069) and *ERF-107* (LOC112033868), were more expressed in CR than in BS samples (Figure 3C). Furthermore, two homologs of the receptor-like kinase *Feronia* were detected in the CR_DE library, and the expression of one of them is shown in Figure 3C (LOC112001600). *Feronia* kinases are involved in ethylene and ABA signaling pathways, among other processes. 

In the case of ABA-related genes, results also suggest a greater content or sensibility of this hormone in mature than in juvenile-like samples. The differential expression of two ABA receptors (*PYL-4*, LOC111988217 and LOC112017749) and the TF *ABSCISIC ACID-INSENSITIVE 5-like protein 3* (*ABI-5*, LOC112026097) supports this idea (Figure 3D). Additionally, the *E3 ubiquitin-protein ligase RGLG5* (LOC112025452) was also more active in CR samples. *RGLG5* is a positive regulator of the ABA response that targets the PP2CA phosphatases, major inhibitors of ABA responses, for degradation, according to the Uniprot annotation (uniprot.org). Overall, these results suggest that both ET and ABA signaling pathways present a higher level of activity in mature than in juvenile-like tissues, thus indicating a potential higher content of both hormones in these shoots.

**Figure 4:**
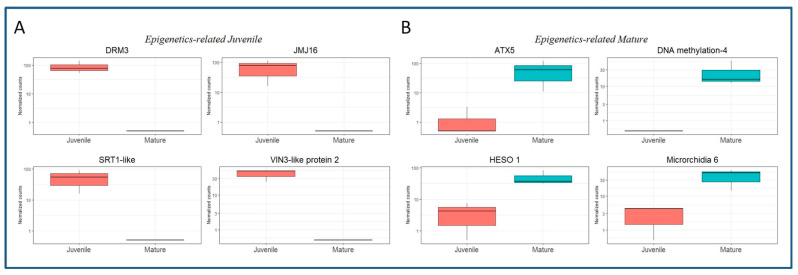
Boxplots showing the expression in normalized counts of DEGs related to epigenetics mechanisms. (**A**) DEGs in the BS samples. *DNA (cytosine-5)-methyltransferase DRM3*, LOC112032791; *Lysine-specific demethylase JMJ16*, LOC112019264; *NAD-dependent protein deacetylase SRT1*, LOC112028975; *VIN-3-like protein 2* (*VIL2*), LOC112021978. (**B**) DEGs in the CR samples. *Histone-lysine N-methyltransferase ATX5*, LOC112017971; *DNA-methylation Factor 4*, LOC111987241; *HESO-1*, LOC111999674; *Microrchidia 6*, LOC111996263.

Several DEGs related to epigenetics processes were detected in both BS_DE and CR_DE libraries, and the available information concerning their role is provided in the Uniprot database. The expression of eight of the identified DEGs is shown in Figure 4. Expression of *DNA (cytosine-5)-methyltransferase DRM3* (DRM3, LOC112032791, involved in methylation and small interfering RNA production); *Lysine-specific demethylase JMJ16* (*JMJ16*, LOC112019264); *NAD-dependent protein deacetylase SRT1* (*SRT1*, LOC112028975); and *VIN-3-like protein 2* (*VIL2*, LOC112021978, involved in histone binding), was detected in BS but not in CR samples (Figure 4A). These results suggest that these genes play a role in the epigenetic reprogramming of rooting-competent cells in BS shoots. On the other hand, DEGs preferentially expressed in CR shoots might be related to epigenetic modifications that preclude the acquisition of rooting competence by specific cells (Figure 4B). Some of the relevant identified genes falling in this category were *DNA-methylation Factor 4* (LOC111987241), *Histone-lysine N-methyltransferase ATX5* (*ATX5*, LOC112017971), *HESO-1* (LOC111999674, involved in the Argonaute-1 mediated degradation of miRNA targeted RNAs) and *Microrchidia 6* (LOC111996263, related to gene silencing by RNA-directed DNA methylation).

To gain further insight into the processes regulated by the identified DEGs, a gene ontology analysis of the CR_DE and BS_DE datasets was performed. Several biological processes were represented in BS DEGs, including Carbohydrate Metabolism, RNA Metabolism and Transmembrane Transport (Figure S2). Cellular Components included Integral Component of Membrane, Nucleus, Chloroplast and Extracellular Region (Figure S2). Finally, the DEGs detected in BS samples are involved in Molecular Functions related to enzymatic activities (Hydrolase, Transferase and Oxidoreductase), and Protein and Nucleic Acid Binding (Figure S2). In the case of mature samples, DEGs were predominantly related to the Biological Processes shown in Figure S3, Protein Phosphorylation, Defense Response, Nucleotide Metabolism and Regulation of Transcription. Interestingly, the lower number of biological processes in CR than in BS samples suggests a more tightly regulated response in mature shoots. Main Cellular Components included Cytoplasm, Nucleus and Integral Component of Membrane (Figure S3). The more relevant Molecular Functions were Hydrolase activity, ATP and Protein Binding, Protein Kinase Activity and Metal Ion Binding (Figure S3). Overall, results suggest that there were significant differences in the GO categories between both conditions that might directly influence the differential responses of both types of shoots to both wounding and auxin.

**Figure 5:**
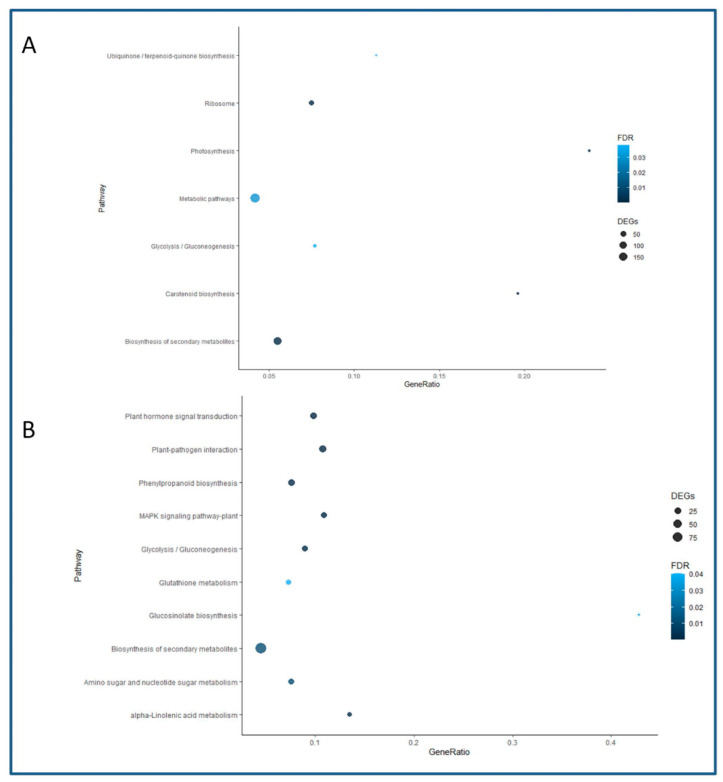
Enriched pathways in the KEGG analysis. The name of the pathway is shown on the *Y* axis, and the proportion of genes identified in the libraries with respect to the total number of genes in the corresponding pathway is given on the *X* axis as Gene Ratio. The size of the dots indicates the number of genes detected, and the color indicates the value for the False Discovery Rate (FDR). (**A**) Enriched pathways in the BS samples. (**B**) Enriched pathways in the CR samples.

A KEGG pathways enrichment analysis was developed to further characterize the responses of BS and CR shoots to the root induction treatment. However, due to the limited amount of *Q. suber* genes annotated in this database, a less stringent approach was chosen (see Materials and Methods). In this way, the number of detected DEGs increased in both types of samples, reaching 1888 for the BS library and 1847 for the CR library. The Biosynthesis of Secondary Metabolites and the Glycolysis/Gluconeogenesis pathways were found to be enriched in both datasets (98 and 20 genes in CR, and 121 and 17 genes in BS, respectively). The rest of the enriched pathways in BS and the number of detected genes included Ribosome (40 genes), Photosynthesis (10 genes) and Carotenoid Biosynthesis (10 genes), among others (Figure 5A). On the other hand, CR-enriched pathways and the corresponding number of genes included Plant Hormone Signal Transduction (27 genes), Plant-Pathogen Interaction (32 genes), Glucosinolate biosynthesis (7 genes), Glutathione (15 genes) and Alpha-linolenic Acid Metabolism (13 genes) (Figure 5B). Due to the relevance of the Plant Hormone Signal Transduction pathway, data obtained for this route were compared between CR and BS samples, although for the latter it was not found to be significantly enriched. A complete list of the DEGs detected for each sample in this pathway is presented in Supplemental Tables S6 and S7. Auxin-related signaling was found to be the most relevant route in both types of shoots; however, more specific hormone signaling routes, including ABA and SA, were enriched in CR than BS samples. On the other hand, CKs-related DEGs were detected in BS samples. Additionally, the preferential expression of two ABA-negative modulators (*Protein Phosphatase 2C*) in juvenile-like tissues suggests that ARs formation is favored by blocking ABA signaling. 

**Figure 6:**
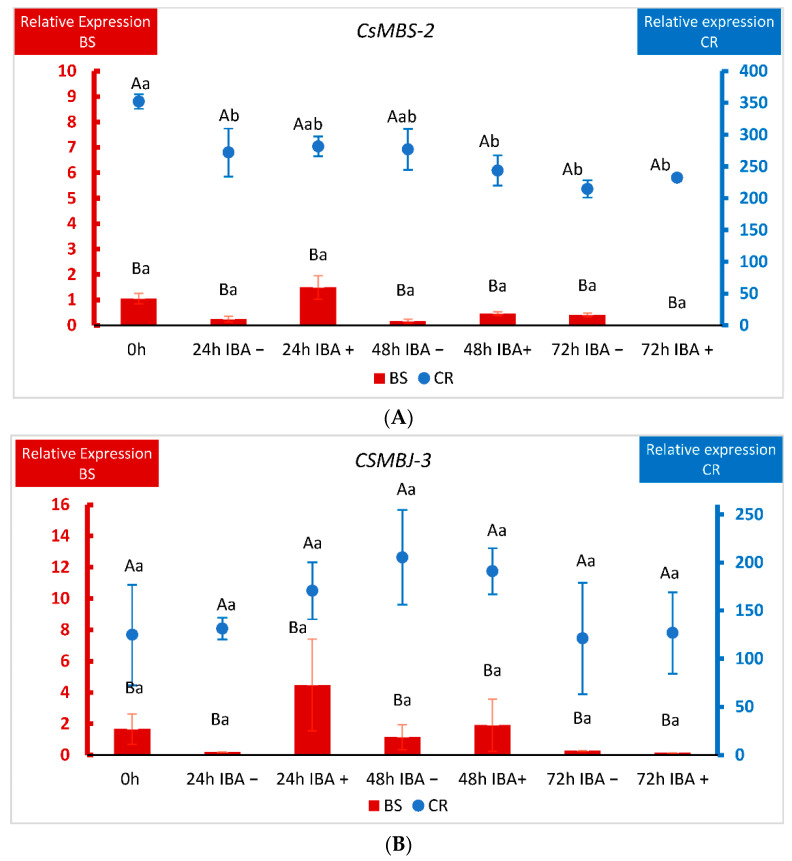

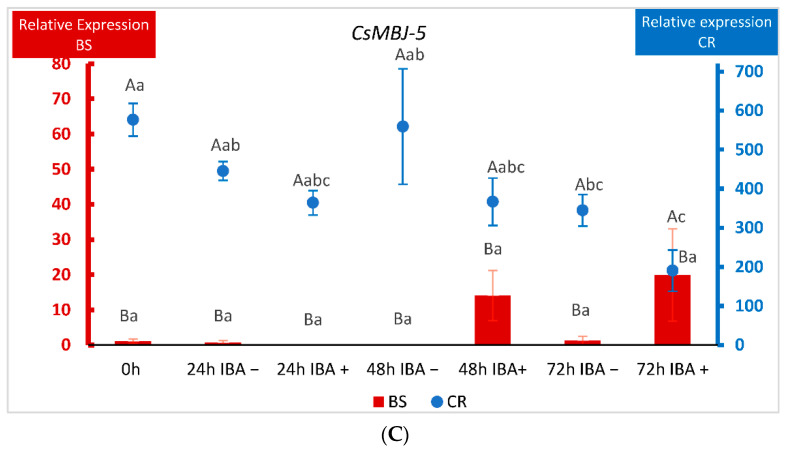
qPCR analysis of the expression of three MADS-box genes in chestnut tissues. (**A**) Expression of the *CsMADS-box SVP-2* gene (*MBS2*) in BS (Juvenile) samples and CR (Mature) samples. (**B**) Expression of the *CsMADS-box Jointless-3* gene (*MBJ3*) in BS (Juvenile) samples and CR (Mature) samples. (**C**) Expression of the *CsMADS-box Jointless-5* gene (*MBJ5*) in BS (Juvenile) samples and CR (Mature) samples. Data correspond to mean ± standard error. 0 h: samples collected at the end of the multiplication cycle and prior to any treatment. Samples were wounded (−) or wounded and treated with IBA (+) and collected at the indicated times (in hours). Due to the differences in magnitude between juvenile and mature lines, BS and CR relative expressions were displayed on different Y-axes. The data were subjected to two-way analysis of variance (ANOVA), followed by the comparison of group means (Tukey-b test), or to the Welch ANOVA, followed by Games–Howell post-hoc comparison (when heteroscedasticity was detected). Different capital letters indicate significant differences (*p* < 0.05) in relation to the line, and different lowercase letters indicate significant differences in relation to the treatment. Due to the significant interaction found in genes *CsMBS-2* and *CsMBJ-5*, Bonferroni’s adjustment was applied to detect simple main effects.

The detection of nine genes from the MADS-box family of TFs in CR shoots prompted us to further characterize their expression. We chose three of the identified genes and analyzed their behavior in BS and CR shoots during the first 72 h after root induction (Figure 6). We performed a two-way ANOVA in which the factors were the line (CR and BS) and the time after wounding or wounding plus IBA application. As explained in Materials and Methods (Section 4.1 and Section 4.5), shoots harvested at time 0 (control) did not receive IBA, which prevented us from studying the effect of time and IBA treatment as separate factors in this experiment. *CsMBS-2*, *CsMBJ-3* and *CsMBJ-5* showed a significantly higher level of expression in CR samples at all time-points analyzed (*p* < 0.001), including at the beginning of the experiment (0 h), while little expression was detected in BS samples (Figure 6A–C).

Due to this difference of magnitude, BS and CR relative expressions were displayed on different Y-axes. In the case of *CsMBS-2*, wounding and wounding plus auxin lowered the levels of the gene in CR shoots, although the expression remained always higher than in shoots derived from BS (Figure 6A). For *CsMBJ-3*, the variations in expression within samples in each line (CR and BS) were not significant (Figure 6B). With respect to *CsMBJ-5*, behavior was also quite different according to the ontogenetic state of the tissues. In mature shoots *CsMBJ-5* showed an extremely high level of expression at the beginning of the experiment that was lowered for all treatments, except 48 h without auxin (Figure 6C). On the other hand, in BS shoots auxin treatment apparently increased the expression of the gene at 48 and 72 h, but these changes were not significant (Figure 6C). Therefore, although the three genes showed different dynamics in response to treatments in BS and CR samples, levels of expression were always much higher in mature shoots. 

In order to further validate the expression profiles acquired from the transcriptomics analysis, the expression of six genes was analyzed by qPCR and compared with the normalized counts obtained in the transcriptomics analysis (Supplementary Figure S4). Trends in the expression of the genes were conserved in both experiments, thus validating the results from the long-read sequencing transcriptomics analysis.

## 3. Discussion

In many Fagaceae species and other tree genera, maturation imposes a severe decline of the morphogenetic ability, thus limiting the vegetative propagation of adult genotypes of these species through ARs formation or the development of somatic embryogenesis protocols [10]. Although many aspects of the molecular mechanisms of adventitious rooting have been unraveled in recent years, particularly in model species [4,11], the molecular nature of recalcitrance is not yet understood [6,10]. In particular, the influence of the ontogenetic state on the molecular responses to auxin and other stimuli is an underrepresented area of study. In the present work, two in vitro lines of chestnut shoots with the same genotype but different ontogenetic state were subjected to IBA treatment. The transcriptomic response was analyzed after 24 h, the moment in which dedifferentiation has already occurred, cellular reprogramming and induction are taking place, and the first cell divisions are detected [7]. These lines have been previously characterized at the physiological and specific gene expression levels, showing contrasting responses, metabolite content and gene behavior (reviewed in [7]). While the juvenile-like line roots successfully, mature shoots only form calluses in response to exogenous auxin. These lines show differences in auxin and polyamines content, as well as in the expression of genes potentially involved in the formation of ARs [16,17,20,21,22]. Previous results suggested that maturation provokes a dramatic change in the physiology of the shoots by altering hormone profiles and gene expression.

The results of the present study showed a significant differential response between both types of shoots that affected many facets of plant physiology including the expression of TFs, genes related to plant hormone signaling, defense response genes and secondary metabolites. Despite the limitations derived from the lack of a reference genome from the same species, the use of the *Q. suber* genome assembly [23] allowed a successful mapping of nearly 80% of the reads from both lines. However, a significant degree of variability within the replicates from each type of lines was detected. Previous analysis of the AR response in chestnut and other related species has shown that it is a highly asynchronous process. The first cellular divisions, the emergence of the new roots in BS shoots or the formation of calluses in mature tissues takes place within a wide timeframe [20,24]. Additionally, the proper heterogeneity of the tissues under analysis might account for the variability. 

In the current analysis, around 15% and 10% of the DEGs from the BS line and CR line, respectively, were described in the databases as Uncharacterized proteins (Supplementary Table S3), suggesting that many genes involved in the responses to auxin and wounding are not yet characterized in these species (*Q. suber* and *C. sativa*). Moreover, a relevant number of DEGs (14 in BS_DE and 25 in CR_DE) described as pseudogenes in the *Q. suber* genome draft were also found. Pseudogenes are considered non-functional versions of active genes that are generated due to the accumulation of degenerative features such as in-frame stop codons and frame-shift or base mutations in the coding region [25]. However, a significant number of pseudogenes are expressed and may operate as RNA genes [26]. In addition, we also detected the expression of several lncRNAs in the responses of chestnut shoots to wounding and auxin (Supplementary Table S3). They are thought to mediate gene expression by different means, including post-transcriptional regulation, DNA methylation, chromatin remodeling or as target mimics of miRNAs [27]. All of these DEGs emerge as putative relevant players in the process of AR or recalcitrant behavior, although more research is needed to clarify their roles.

Several TFs have been identified for their role in AR in model and tree species; however, the identity of other relevant TFs remains elusive. Moreover, how the expression of these TFs shifts in response to maturation is not properly characterized. Three TFs from the GRAS family were identified in the BS_DE dataset (Supplementary Table S4). Members of this family have been previously characterized as being involved in ARs formation in woody species [28]. In previous works, the activity of *CsScarecrow-like-1 (CsSCL-1)*, another GRAS TF, was found to be directly related to the early establishment of RFCs in auxin-treated root competent shoots. The expression of *CsSCL-1* was induced by auxin in specific zones of the vascular bundles and neighboring areas of chestnut juvenile-like shoots where roots are initiated [16]. However, its expression was localized in a wider area in mature shoots. The identification of three GRAS genes differentially expressed in BS samples (two *SCL-3* and one *SCL-7*) reinforces the hypothesis that *Scarecrow-like* genes are positively related to the acquisition of rooting competence in BS shoots, and they may act in a redundant or cooperative fashion controlling gene expression related to AR. 

Three Ethylene Response Factors (ERFs) were differentially expressed in mature shoots, *ERF98*, *ERF107* and *RAP-2.3* (Supplementary Table S5), while one was detected in the BS shoots (*RAP2.7*, Supplementary Table S4), suggesting that ET might be involved in the initial stages of the formation of both ARs and calluses, although to a distinct degree. *RELATED to APETALA* (*RAP*) genes belong to the ERF-VII subgroup of the AP2/ERF superfamily, and their activity has been linked to stress responses such as low oxygen and osmotic and oxidative stress, inducing a metabolic reprogramming to prevent tissue damage under those conditions [29,30]. In a previous work, the expression of a member of this subgroup, *RAP2.12*, was analyzed during the induction of ARs in chestnut and oak (*Quercus robur*). The expression of the gene was induced in juvenile-like and mature shoots of both species treated or not with IBA, implying its relation to wounding responses [22]. However, tissue-specific expression of *CsRAP2.12* was detected in the cambial region only in chestnut BS shoots, suggesting a positive relationship of tissue-specific expression with AR [22]. On the other hand, differential expression of *ERF98* and *ERF107* in mature shoots might be related to the formation of calluses in the CR line. Studies in Arabidopsis have shown that *AtERF115* is involved in tissue regeneration after wounding, locally restricting auxin signaling within the damaged zone [31]. Nonetheless, *ERF115* has also been shown to prevent AR through the integration of JA and CK cues. It is expressed in vascular tissues, where it modulates CK homeostasis, and its inhibiting activity cannot be overturned by auxin [32]. Whether chestnut *ERF98* and *ERF107* genes also integrate JA- or CK-signaling to inhibit AR is an interesting possibility that might help explain recalcitrant behavior of CR shoots.

The differential expression of several epigenetics-related genes in both types of shoots (Figure 4) suggests that morphogenetic responses in chestnut are under epigenetic control that putatively integrates other cues such as maturation or phytohormone signaling. The expression of *SRT1* in juvenile tissues could be linked to the ET effect in AR. In Arabidopsis, *AtSRT1* has been shown to repress the expression of several ethylene-responsive genes via histone acetylation [33]. Therefore, the above-mentioned inhibitory effect of ET in AR might be circumvented in juvenile tissues through an epigenetics-based mechanism. On the other hand, the presence of DEGs involved in gene silencing in CR shoots (*DNA methylation factor 4* and *Microrchidia 6*) might be blocking the expression of specific genes in these tissues, and this block cannot be reversed by the induction treatment. Gene ontology and KEGG analysis suggest that multiple pathways are involved in the differential response between both types of shoots, and therefore maturation might exert a major remodeling of chestnut physiological performance. In the Biological Processes category, signaling processes, defense response and DNA-related activity were found in the CR samples. However, a greater number of categories were detected in BS than in CS shoots, including several metabolic processes, RNA metabolism and transmembrane transport. The observed differences suggest that a complex orchestrated response is activated in juvenile-like shoots, leading to the formation of ARs, while in CR shoots activity related to signaling and defense responses prevail. Moreover, chloroplast-related activity was higher in BS than in CR samples, while several molecular functions were shared between BS and CR shoots. Both libraries were enriched in the Glycolysis/Gluconeogenesis pathway, suggesting that AR and callus formation are high energy-demanding processes. Wounding and auxin induce the local accumulation of carbohydrates in cuttings, in order to provide the energy needed for the correct repair of the damaged tissues (reviewed in [3]), and the chestnut data support this idea. The biosynthesis of secondary metabolites pathway was also enriched in both types of tissues; however, BS samples were also enriched in carotenoid biosynthesis, while CR samples showed the presence of the Phenylpropanoid pathway, suggesting specific differences in the secondary metabolites related to each sample. On the other hand, BS samples showed enrichment in the Photosynthesis and Ribosome pathways. Similar results have been found in apple rootstocks and mulberry hardwood cuttings during the formation of ARs [34,35], indicating a link between the expression of photosynthesis-related genes and the formation of ARs, while ribosomal activity might be necessary for the synthesis of specific structural and signaling proteins needed in the process. Furthermore, the analysis of the AR response of walnut shoots revealed an increased activity of a gene module related to photosynthesis in rejuvenated tissues when compared to mature shoots [36]. On the other hand, enriched pathways in CR samples might point to negative relation to AR formation, although contrasting results have been reported in the literature. For instance, enrichment in the glutathione and the alpha-linolenic acid metabolic routes were identified in mature tissues; however, both these routes have been positively linked to AR in apple rootstocks [34], while the latter was also related to AR in tea nodal cuttings [37]. During the study of tissue dedifferentiation in *Camellia sinensis*, the glutathione pathway was reported to be positively related to AR [38]. These contradictory results suggest that the influence of these two routes in morphogenetic processes depends on the maturation state of the tissues. However, different sampling times were applied in each of these studies; therefore, providing data from different AR stages could explain the differences found.

Interestingly, the Glucosinolate and the Phenylpropanid biosynthesis pathways were found to be enriched in CR samples. Glucosinolates are nitrogen- and sulfur-containing compounds related to defense processes, and they are mainly found in Brassicaceae species. Recently, an analysis of glucosinolate-deficient mutants in Arabidopsis revealed a direct relation to the formation of lignin [39]. Together with the generation of lignin-related compounds in the phenylpropanoid route, these results indicate that lignin biosynthesis is promoted in mature tissues in response to wounding and IBA treatment, which might preclude the formation of RFCs in CR shoots. This finding supports the expected opposite nature of xylogenesis and AR formation in cuttings of forest species [19,40]. Moreover, ET has been shown to positively interact with IBA in the induction of metaxylem in Arabidopsis hypocotyls [41]. Together with the ET-responsive TF expression detected, these results suggest a negative role for this hormone in the formation of ARs in chestnut.

The Plant Hormone Signal transduction pathway was enriched in CR samples. However, we also carefully inspected the DEGs within the BS samples in this route, because of its direct impact in the process under analysis (Supplementary Tables S6 and S7). In the CR samples, several auxin-related genes were found, as expected. This included *Small Auxin Upregulated RNAs* (*SAURs*), a *GH3-1* gene and *Aux/IAA* genes. The differential expression of a *GH3* gene in mature tissues had already been detected in a previous work [17]. The Aux/IAA proteins work as repressors of ARFs at low auxin levels, but when auxin concentration increases they bind to the SCF^TIR1∕AFB^ complex and are degraded by the 26S proteasome, allowing the activity of the auxin response factors (reviewed in [42]). In BS samples, the expression of the auxin receptor *TIR1* and two *SAURs* was detected, but no *Aux/IAA* genes were identified. The differential expression of *TIR1* suggests that in BS shoots Aux/IAA proteins are degraded. Therefore, although auxin-related signaling was induced in both types of shoots, the specific response in each case was notably different, probably as a consequence of a different level of auxin sensibility. Moreover, specific genes related to ABA and SA signaling were found in CR shoots 24 h after treatment, suggesting a negative relation to the acquisition of rooting competence, which is in agreement with previous results in cuttings of other species [15]. On the other hand, the role of ET and JA seems to be less determinant, at least at the signaling level. A very limited number of DEGs could be related to either one or the other phytohormone, although results from the TFs (Supplementary Table S5) suggest that ET might have a more significant role in callus formation, at least in the induction phase. For JA, the expression of a *MYC2* homolog (LOC112027257) was found in BS samples. *MYC2* is a master regulator of JA-signaling, and its expression 24 h after AR induction has been described in *Populus ussuriensis* [43]. Interestingly, some CK-signaling DEGs were detected in BS samples, suggesting that these phytohormones might be necessary for the control of the initial cell divisions just after priming of the RFCs.

Within the CR_DE library, nine DEGs from the MADS-box family corresponding to functional genes were detected, while no expression was found in BS tissues (Figure 3A, Supplementary Table S2). Seven of these genes are annotated as *Jointless-like* in the *Q. suber* genome, while two are annotated as *SVP*. The MADS-box superfamily of TFs is related to almost every aspect of morphogenesis in all phases of the plant life cycle, with a distinguished role in the transition to flowering, although they have also been linked to abiotic stress and plastic developmental responses [44,45]. In this and other processes, MADS-box genes provide a combinatorial code in which the physical interaction between different MADS-box proteins results in the formation of homo- and heterodimers, or tetramers. Therefore, distinct complexes might control the expression of common or specific genes, according to the identity of the involved proteins [46]. Thus, the MADS-box genes identified here emerge as putative candidates controlling transcriptional responses in mature tissues that preclude the acquisition of rooting competence in these shoots. qPCR analysis of three of the identified MADS-box genes, two *Jointless-like* and one *SVP*, showed that their level of expression is much higher in CR shoots than in BS shoots from the beginning of the experiment. Although wounding and auxin treatment might modify their expression profile, levels remained higher in mature tissues. In olive trees or citrus species, several MADS-box genes showed higher expression in mature than in juvenile tissues [47,48]. According to homology analysis (results not shown), *Jointless* and *SVP* genes fall within the StMADS11 subfamily, whose members are believed to be preferentially expressed in vegetative tissues [49]. In peach and apple, *DAM* MADS-box genes, closely related to *SVP*, integrate phytohormone and environmental signals to control bud dormancy, and a possible link with the *Squamosa* gene *MdSPL9* has been proposed in apple [50,51]. Moreover, ABA-controlled expression of a *Jointless* gene and an *SVP* gene in peach and aspen, respectively, has been described [45,52], and ABA content in pear was found to be greater in mature tissues [53]. Therefore, activity of these TFs might be impeding the necessary reprogramming of cells in the mature shoots. Moreover, the prevalence of ABA signaling in CR shoots, as seen from the DEGs detected in the CR_DE library, could be one of the factors influencing the expression of MADS-box genes in mature tissues. A negative relationship of ABA with the ability to form ARs has been shown in Arabidopsis hypocotyls and apple [54,55]. On the other hand, the activity of two MADS-box genes, *VCM1* and *VCM2*, has been linked to vascular cambium development in poplar. These genes seem to control the expression of an intracellular auxin transporter, *PIN5b*, resulting in a reduction of cytosolic auxin and subsequently in a low proliferation activity of the cambium and less xylem differentiation [56]. However, these genes show limited homology with the *Jointless* genes identified in chestnut. Although the identity of the genes controlled by *MADS-box Jointless* and *SVP* TFs is unknown, the presence of three NAC TFs in the CR_DE group is an interesting result. NAC TFs are known to be the master regulators of xylogenesis, and their expression might be linked to the process of xylem differentiation in mature tissues [28,57]. As mentioned above, AR and xylogenesis are known to be mutually exclusive in response to IBA [41]; therefore, a putative module comprising Jointless and NAC TFs might be controlling the response to IBA in CR shoots driving the xylem differentiation that results in the formation of calluses. Overall, the differential expression of the nine *MADS-box* genes in mature tissues suggests their possible relation to the integration of different cues, such as ABA and the ontogenetic state, into a gene regulatory network that controls developmental responses and might exert an inhibitory effect on the formation of ARs, while promoting the formation of calluses. 

## 4. Materials and Methods

### 4.1. Plant Material and Root Induction

Chestnut (*Castanea sativa* Mill.) shoot explants were established from an adult field-grown tree and have been kept in vitro for 30 years. Basal sprouts (BS) taken from the base of the trunk present a juvenile-like character, while shoots derived from crown branches (CR) exhibit mature features. Particularly, shoots from the BS line root satisfactorily when treated with exogenous auxin (rooting rate 90%) while CR explants do not form roots but rather calluses when subjected to the same treatment (reviewed in [7]). Shoots were subcultured in vitro under a 4-week regime in GD culture medium [58] supplemented with 0.44 μM 6-benzyladenine (BA), 30 g L^–1^ sucrose and 0.7% Difco Agar. At the end of the multiplication cycle, 3 cm-long shoots were used for the rooting experiments. For root induction, the lower part of the stem was removed and the basal end of the shoots was dipped in an IBA (4.9 mM) solution for 60 s, and the shoots were transferred to 300-mL jars containing 50 mL of hormone-free GD medium (1/3 macronutrient strength). Three biological replicates of the treatment for the BS line (BS01, BS02, BS03) and the CR line (CR01, CR02, CR03) were developed.

For qPCR analysis of the expression of the MADS-box genes, samples from BS and CR shoots were collected at the end of the multiplication cycle (Time 0, Ctrl), and at 24, 48 and 72 h after they were wounded and treated (+) or not (−) with auxin.

### 4.2. RNA Extraction and Quality Assessment

For transcriptomics analysis, 24 h after root induction treatment, the basal parts of the shoots (1 cm, N = 15) were collected and frozen in liquid nitrogen. Total RNA was extracted as described by [59]. After DNAase I treatment, RNA quality of the six samples was evaluated with a Nanodrop 2000c spectrophotometer and a Qubit 4 fluorometer (Thermo Fisher Scientific, Waltham, MA, USA). Samples that met the expected quality criteria were used for the development of the transcriptomics libraries. For the qPCR experiment, the same extraction procedure and type of samples were used and collected at the indicated times (Time 0, 24 h, 48 h and 72 h). Expression values are given relative to the level of BS Ctrl replicate 1.

### 4.3. Sequencing and Bioinformatics Analysis

Sequencing was performed on a MinION device (Oxford Nanopore Technologies, ONT, Oxford, UK) using Flow Cells R9.4.1. The transcriptomics libraries were created with the PCR-cDNA Barcoding kit (SQK-PCB109, ONT), using total RNA and following the manufacturer’s instructions. Briefly, RNAs were reverse-transcribed and strand-switched, amplified by PCR (14 cycles) and specific barcodes for each library were added. Afterwards, adapters were attached to the transcripts and equal molar quantities of the six libraries were pooled together and loaded in the flow cell attached to the MinION device. The sequencing run was developed for 40 h. The software Guppy (v3.3.1) was used for basecalling and demultiplexing. The quality of the reads was initially evaluated with the Epi2me software (Metrichor Ltd., Oxford, UK). The obtained reads were trimmed with Porechop (github.com/rrwick/Porechop, accessed on 21 April 2021), and the quality was evaluated again with FastQC [60]. 

During the development of this study, no reference genome with the necessary Gene File Format (GFF) file was available from any chestnut species, and therefore the genome from *Quercus suber*, a closely related species, was used [23]. The reads obtained in the sequencing experiment were aligned and mapped against this genome with Minimap2 [61], with the option -ax splice. The transcriptomes from the six libraries were assembled and quantified with Stringtie 2 [62], as this version enables the use of reads from long-read sequencing experiments and corrects for the expected error rate. Mapping and assembly of specific transcripts was visually inspected with the Integrative Genomics Viewer [63].

### 4.4. Differential Gene Expression, GO and KEGG Analysis

To detect differentially expressed genes (DEGs) between conditions (maturation state), the DESeq2 software within the R environment was used [64]. Benjamini and Hochberg´s adjustment of the resulting p values was used to establish the False Discovery Rate (FDR), set at *p* < 0.1. Transcripts with a log2 Fold Change > 0 were identified as DEGs in the CR library, while those with a log2 Fold Change < 0 were identified as DEGs in the BS library, forming the respective differentially expressed datasets (CR_DE and BS_DE, respectively). Since the *Q. suber* genome was used as reference, the corresponding gene symbols for each homolog in this species are given in the datasets and in the results for clarity purposes. The two datasets were then used independently for a Gene Ontology analysis. The software Blast2GO was used to identify enriched terms for Biological Process, Molecular Function and Cellular Component, and presented as Treemaps. Regarding the KEGG analysis (Kyoto Encyclopedia of Genes and Genomes), only a limited number of genes are annotated to pathways in the *Q. suber* genome. Therefore, in order to collect a significant amount of information, a less stringent approach was used for the identification of DEGs, setting the FDR to 0.1 for not adjusted *p* values, and the log2 Fold Change to >1 and <1 for CR and BS libraries, respectively. By these means, 1847 and 1888 transcripts were included in the CR and BS datasets, respectively, providing the basis for the identification of a higher number of enriched pathways in both libraries. KEGG analysis was developed with the Kobas tool [65], and the statistical enrichment of DEGs in the different pathways was calculated with the hypergeometric and the Fisher´s exact tests (*p* value < 0.05, corrected *p* value < 0.05).

### 4.5. qRT-PCR

Three members from the group of MADS-box genes identified for being over-expressed in the CR library were selected for further expression characterization. For this analysis, samples of BS and CR shoots were collected at the end of the multiplication cycle (Ctrl, Time 0). Additionally, samples were collected at 24, 48 and 72 h after shoots were treated (+) or not (−) with IBA. Only the basal part of the shoots (1 cm) was used. Total RNA was extracted using the FavorPrep “Total RNA Purification Mini Kit (Woody Plant)” from Favorgen. The genes chosen were *CsMBS2* (*Castanea sativa MADS-box Short Vegetative Phase 2*, homolog of LOC112123007), *CsMBJ3* (*Castanea sativa MADS-box Jointless 3*, homolog of LOC111999454) and *CsMBJ5* (*Castanea sativa MADS-box Jointless 5*, homolog of LOC111999342). *Actin-2*, *Elongation Factor-1* and *Tubulin* were used as internal reference genes. The sequences of primers used in this study are listed in Supplemental Table S8. Reactions were carried out in a final volume of 20 µL, and 12.5 nanograms of cDNA were used for each reaction. The relative expression level was calculated by the Comparative Ct Method [66], and DataAssist v3.0 software (Applied Biosystems, Thermo Fisher Scientific, Waltham, MA, USA) was used for global normalization. The data were subjected to two-way analysis of variance (ANOVA) followed by the comparison of group means (Tukey-b test) or to the Welch ANOVA followed by the Games–Howell post-hoc comparison (when heteroscedasticity was detected). When an interaction between two factors was indicated by the two-way ANOVA, Bonferroni’s adjustment was applied to detect the simple main effects in multiple post-hoc comparisons. Statistical analyses were performed using SPSS 27.0 (IBM, Armonk, New York, NY, USA).

To further validate the results from the transcriptomics analysis, another six genes were randomly selected and their expression was analyzed by quantitative real-time PCR in the same samples used for transcriptomics. All experiments were performed as previously described [17,22]. 

## 5. Conclusions

The transcriptomics analysis performed in chestnut BS and CR shoots 24 h after the induction treatment (wounding + IBA) provided relevant information about the differences in the gene expression modulation that drive their distinct morphogenetic responses. Most significantly, the results of this study indicate that within the first 24 h after the inductive treatment the fate of the process is already determined, suggesting that molecular events within a narrow time frame outline whether a rooting response is developed or not in chestnut shoots. The identification of a relevant number of *MADS-box* TFs expressed in mature tissues suggest that they might work integrating information from several cues, putatively linked to a higher content of ABA, SA and ET in mature shoots that is related to their recalcitrant behavior. In BS samples, induction of photosynthesis-related genes and specific TFs, like those from the GRAS family, enables the dedifferentiation and determination of specific cells to form RFCs. As expected, IBA induces different molecular routes according to the ontogenetic state of the tissues, giving way to the formation of ARs in juvenile-like tissues while xylogenesis is activated in mature tissues. These results open several potential approaches to tackle recalcitrance in chestnut and to develop improved protocols for the vegetative propagation of selected material, as well as enhancing our understanding of the mechanisms involved in the acquisition of rooting competence and their links to maturation.

## Data Availability

The FastQ files generated for each of the samples have been submitted to the NCBI repository (https://www.ncbi.nlm.nih.gov/bioproject, accessed on 18 April 2022) under accession ID PRJNA824049.

## Supplementary Materials

The following supporting information can be downloaded at: https://www.mdpi.com/article/10.3390/plants11243486/s1, Figure S1: Integrative Genome Viewer screenshots; Figure S2: Treemaps of the BLAST2GO Ontology analysis of the BS_DE library; Figure S3: Treemaps of the BLAST2GO Ontology analysis of the CR_DE library; Figure S4: qPCR validation; Table S1: list of DEGs in the BS_DE library; Table S2: list of DEGs in the CR_DE library; Table S3: number of Uncharacterized genes, Pseudogenes and long non-coding RNAs (lncRNAs) in the BS_DE and CR_DE libraries; Table S4: transcription factors detected in the BS_DE library; Table S5: transcription factors detected in the CR_DE library; Table S6: list of genes related to Plant Hormone Signaling Pathways in the BS samples; Table S7: list of genes related to Plant Hormone Signaling Pathways in the CR samples; Table S8: primers used for qPCR analysis of the expression of the MADS-box genes and the qPCR validation.

## References

[1] Steffens B., Rasmussen A. (2016). The Physiology of Adventitious Roots. Plant Physiol..

[2] Díaz-Sala C. (2014). Direct reprogramming of adult somatic cells toward adventitious root formation in forest tree species: The effect of the juvenile to adult transition. Front. Plant Sci..

[3] Druege U., Hilo A., Pérez-Pérez J.M., Klopotek Y., Acosta M., Shahinnia F., Zerche S., Franken P., Hajirezaei M.R. (2019). Molecular and physiological control of adventitious rooting in cuttings: Phytohormone action meets resource allocation. Ann. Bot..

[4] Guan L., Murphy A.S., Peer W.A., Gan L., Li Y., Cheng Z.-M. (2015). Physiological and Molecular Regulation of Adventitious Root Formation. CRC. Crit. Rev. Plant Sci..

[5] Legué V., Rigal A., Bhalerao R.P. (2014). Adventitious root formation in tree species: Involvement of transcription factors. Physiol. Plant..

[6] Abarca D. (2021). Identifying Molecular Chechkpoints for Adventitious Root Induction: Are We Ready to Fill the Gaps?. Front. Plant Sci..

[7] Vielba J.M., Vidal N., José M.C.S., Rico S., Sánchez C. (2020). Recent Advances in Adventitious Root Formation in Chestnut. Plants.

[8] Da Costa C.T., de Almeida M.R., Ruedell C.M., Schwambach J., Maraschin F.S., Fett-Neto A.G. (2013). When stress and development go hand in hand: Main hormonal controls of adventitious rooting in cuttings. Front. Plant Sci..

[9] Druege U., Franken P., Hajirezaei M.R. (2016). Plant Hormone Homeostasis, Signaling, and Function during Adventitious Root Formation in Cuttings. Front. Plant Sci..

[10] Díaz-Sala C. (2019). Molecular Dissection of the Regenerative Capacity of Forest Tree Species: Special Focus on Conifers. Front. Plant Sci..

[11] Gonin M., Bergougnoux V., Nguyen T.D., Gantet P., Champion A. (2019). What Makes Adventitious Roots?. Plants.

[12] Han S., Hwang I. (2018). Integration of multiple signaling pathways shapes the auxin response. J. Exp. Bot..

[13] Ahkami A.H., Lischewski S., Haensch K., Porfirova S., Hofmann J., Rolletschek H., Melzer M., Franken P., Hause B., Druege U. (2009). Molecular physiology of adventitious root formation in Petunia hybrida cuttings: Involvement of wound response and primary metabolism. New Phytol..

[14] Moore B.M., Lee Y.S., Wang P., Azodi C., Grotewold E., Shiu S.-H. (2022). Modeling temporal and hormonal regulation of plant transcriptional response to wounding. Plant Cell.

[15] Bannoud F., Bellini C. (2021). Adventitious Rooting in Populus Species: Update and Perspectives. Front. Plant Sci..

[16] Vielba J.M., Diaz-Sala C., Ferro E., Rico S., Lamprecht M., Abarca D., Ballester A., Sanchez C. (2011). *CsSCL1* is differentially regulated upon maturation in chestnut microshoots and is specifically expressed in rooting-competent cells. Tree Physiol..

[17] Vielba J.M., Varas E., Rico S., Covelo P., Sánchez C. (2016). Auxin-mediated expression of a GH3 gene in relation to ontogenic state in Chestnut. Trees.

[18] Pizarro A., Díaz-Sala C. (2019). Cellular dynamics during maturation-related decline of adventitious root formation in forest tree species. Physiol. Plant..

[19] Pizarro A., Díaz-Sala C. (2020). Effect of polar auxin transport and gibberellins on xylem formation in pine cuttings under adventitious rooting conditions. Isr. J. Plant Sci..

[20] Ballester A., San-José M.C., Vidal N., Fernández-Lorenzo J.L., Vieitez A.M. (1999). Anatomical and Biochemical Events during in vitro Rooting of Microcuttings from Juvenile and Mature Phases of Chestnut. Ann. Bot..

[21] Sanchez C., Vielba J.M., Ferro E., Covelo G., Sole A., Abarca D., de Mier B.S., Diaz-Sala C. (2007). Two SCARECROW-LIKE genes are induced in response to exogenous auxin in rooting-competent cuttings of distantly related forest species. Tree Physiol..

[22] Valladares S., Varas E., Vielba J.M., Vidal N., Codesido V., Castro R., Sanchez C. (2020). Expression of a *Rap2.12 like-1* ERF gene during adventitious rooting of chestnut and oak microshoots. Isr. J. Plant Sci..

[23] Ramos A.M., Usié A., Barbosa P., Barros P.M., Capote T., Chaves I., Simões F., Abreu I., Carrasquinho I., Faro C. (2018). The draft genome sequence of cork oak. Sci. Data.

[24] Vidal N., Arellano G., San-Jose M.C., Vieitez A.M., Ballester A. (2003). Developmental stages during the rooting of in-vitro-cultured *Quercus robur* shoots from material of juvenile and mature origin. Tree Physiol..

[25] Garewal N., Goyal N., Pathania S., Kaur J., Singh K. (2021). Gauging the trends of pseudogenes in plants. Crit. Rev. Biotechnol..

[26] Xie J., Chen S., Xu W., Zhao Y., Zhang D. (2019). Origination and Function of Plant Pseudogenes. Plant Signal. Behav..

[27] Long Y., Wang X., Youmans D.T., Cech T.R. (2017). How do lncRNAs regulate transcription?. Sci. Adv..

[28] Diaz-Sala C. (2020). A Perspective on Adventitious Root Formation in Tree Species. Plants.

[29] Papdi C., Pérez-Salamó I., Joseph M.P., Giuntoli B., Bögre L., Koncz C., Szabados L. (2015). The low oxygen, oxidative and osmotic stress responses synergistically act through the ethylene response factor VII genes *RAP2.12*, *RAP2.2* and *RAP2.3*. Plant J..

[30] Giuntoli B., Shukla V., Maggiorelli F., Giorgi F.M., Lombardi L., Perata P., Licausi F. (2017). Age-dependent regulation of ERF-VII transcription factor activity in *Arabidopsis thaliana*. Plant. Cell Environ..

[31] Hoermayer L., Montesinos J.C., Marhava P., Benková E., Yoshida S., Friml J. (2020). Wounding-induced changes in cellular pressure and localized auxin signalling spatially coordinate restorative divisions in roots. Proc. Natl. Acad. Sci. USA.

[32] Lakehal A., Dob A., Rahneshan Z., Novák O., Escamez S., Alallaq S., Strnad M., Tuominen H., Bellini C. (2020). ETHYLENE RESPONSE FACTOR 115 integrates jasmonate and cytokinin signaling machineries to repress adventitious rooting in Arabidopsis. New Phytol..

[33] Zhang F., Wang L., Ko E.E., Shao K., Qiao H. (2018). Histone Deacetylases SRT1 and SRT2 Interact with ENAP1 to Mediate Ethylene-Induced Transcriptional Repression. Plant Cell.

[34] Tahir M.M., Chen S., Ma X., Li S., Zhang X., Shao Y., Shalmani A., Zhao C., Bao L., Zhang D. (2021). Transcriptome analysis reveals the promotive effect of potassium by hormones and sugar signaling pathways during adventitious roots formation in the apple rootstock. Plant Physiol. Biochem..

[35] Cao X., Du W., Shang C., Shen Q., Liu L., Cheng J. (2018). Comparative transcriptome reveals circadian and hormonal control of adventitious rooting in mulberry hardwood cuttings. Acta Physiol. Plant..

[36] Song X., Liu H., Bu D., Xu H., Ma Q., Pei D. (2021). Rejuvenation remodels transcriptional network to improve rhizogenesis in mature Juglans tree. Tree Physiol..

[37] Cheng L., Liu H., Zhao J., Dong Y., Xu Q., Yu Y. (2021). Hormone Orchestrates a Hierarchical Transcriptional Cascade That Regulates Al-Induced De Novo Root Regeneration in Tea Nodal Cutting. J. Agric. Food Chem..

[38] Gao Y., Zhao M., Wu X.-H., Li D., Borthakur D., Ye J.-H., Zheng X.-Q., Lu J.-L. (2019). Analysis of Differentially Expressed Genes in Tissues of *Camellia sinensis* during Dedifferentiation and Root Redifferentiation. Sci. Rep..

[39] Hixson K.K., Marques J.V., Wendler J.P., McDermott J.E., Weitz K.K., Clauss T.R., Monroe M.E., Moore R.J., Brown J., Lipton M.S. (2021). New Insights into Lignification via Network and Multi-Omics Analyses of Arogenate Dehydratase Knock-Out Mutants in *Arabidopsis thaliana*. Front. Plant Sci..

[40] Ricci A., Rolli E., Brunoni F., Dramis L., Sacco E., Fattorini L., Ruffoni B., Díaz-Sala C., Altamura M.M. (2016). 1,3-di(benzo[d]oxazol-5-yl)urea acts as either adventitious rooting adjuvant or xylogenesis enhancer in carob and pine microcuttings depending on the presence/absence of exogenous indole-3-butyric acid. Plant Cell Tissue Organ. Cult..

[41] Fattorini L., Della Rovere F., Andreini E., Ronzan M., Falasca G., Altamura M. (2017). Indole-3-Butyric Acid Induces Ectopic Formation of Metaxylem in the Hypocotyl of Arabidopsis thaliana without Conversion into Indole-3-Acetic Acid and with a Positive Interaction with Ethylene. Int. J. Mol. Sci..

[42] Li S.-B., Xie Z.-Z., Hu C.-G., Zhang J.-Z. (2016). A Review of Auxin Response Factors (ARFs) in Plants. Front. Plant Sci..

[43] Wei M., Liu Q., Wang Z., Yang J., Li W., Chen Y., Lu H., Nie J., Liu B., Lv K. (2020). PuHox52-mediated hierarchical multilayered gene regulatory network promotes adventitious root formation in *Populus ussuriensis*. New Phytol..

[44] Smaczniak C., Immink R.G.H., Angenent G.C., Kaufmann K. (2012). Developmental and evolutionary diversity of plant MADS-domain factors: Insights from recent studies. Development.

[45] Castelán-Muñoz N., Herrera J., Cajero-Sánchez W., Arrizubieta M., Trejo C., García-Ponce B., de la P. (2019). Sánchez, M.; Álvarez-Buylla, E.R.; Garay-Arroyo, A. MADS-Box Genes Are Key Components of Genetic Regulatory Networks Involved in Abiotic Stress and Plastic Developmental Responses in Plants. Front. Plant Sci..

[46] Alvarez-Buylla E.R., García-Ponce B., Sánchez M.P., Espinosa-Soto C., García-Gómez M.L., Piñeyro-Nelson A., Garay-Arroyo A. (2019). MADS-box genes underground becoming mainstream: Plant root developmental mechanisms. New Phytol..

[47] García-López M.C., Vidoy I., Jiménez-Ruiz J., Muñoz-Mérida A., Fernández-Ocaña A., de la Rosa R., Barroso J.B., Navarro F., Trelles O., Beuzón C.R. (2014). Genetic changes involved in the juvenile-to-adult transition in the shoot apex of *Olea europaea* L. occur years before the first flowering. Tree Genet. Genomes.

[48] Castillo M.-C., Forment J., Gadea J., Carrasco J.L., Juarez J., Navarro L., Ancillo G. (2013). Identification of transcription factors potentially involved in the juvenile to adult phase transition in Citrus. Ann. Bot..

[49] Khan M.R., Ali G.M. (2013). Functional evolution of cis-regulatory modules of STMADS11 superclade MADS-box genes. Plant Mol. Biol..

[50] Jiménez S., Lawton-Rauh A.L., Reighard G.L., Abbott A.G., Bielenberg D.G. (2009). Phylogenetic analysis and molecular evolution of the dormancy associated MADS-box genes from peach. BMC Plant Biol..

[51] Silveira Falavigna V., Severing E., Lai X., Estevan J., Farrera I., Hugouvieux V., Revers L.F., Zubieta C., Coupland G., Costes E. (2021). Unraveling the role of MADS transcription factor complexes in apple tree dormancy. New Phytol..

[52] Worarad K., Xie X., Martha Rumainum I., Burana C., Yamane K. (2017). Effects of Fluridone Treatment on Seed Germination and Dormancy-associated Gene Expression in an Ornamental Peach (*Prunus persica* (L.) Batsch). Hortic. J..

[53] Song M., Wang R., Zhou F., Wang R., Zhang S., Li D., Song J., Yang S., Yang Y. (2020). SPLs-mediated flowering regulation and hormone biosynthesis and signaling accompany juvenile-adult phase transition in Pyrus. Sci. Hortic..

[54] Zeng Y., Verstraeten I., Trinh H.K., Heugebaert T., Stevens C.V., Garcia-Maquilon I., Rodriguez P.L., Vanneste S., Geelen D. (2021). Arabidopsis Hypocotyl Adventitious Root Formation Is Suppressed by ABA Signaling. Genes.

[55] Tahir M.M., Li S., Liu Z., Fan L., Tang T., Zhang X., Mao J., Li K., Khan A., Shao Y. (2022). Different miRNAs and hormones are involved in PEG-induced inhibition of adventitious root formation in apple. Sci. Hortic..

[56] Zheng S., He J., Lin Z., Zhu Y., Sun J., Li L. (2021). Two MADS-box genes regulate vascular cambium activity and secondary growth by modulating auxin homeostasis in Populus. Plant Commun..

[57] Fukuda H. (2016). Signaling, transcriptional regulation, and asynchronous pattern formation governing plant xylem development. Proc. Japan Acad. Ser. B.

[58] Gresshoff P.M., Doy C.H. (1972). Development and differentiation of haploid *Lycopersicon esculentum* (tomato). Planta.

[59] Chang S., Puryear J., Cairney J. (1993). A simple and efficient method for isolating RNA from pine trees. Plant Mol. Biol. Report..

[60] Andrews S. (2010). FastQC: A Quality Control Tool for High Throughput Sequence Data. http://www.bioinformatics.babraham.ac.uk/projects/fastqc/.

[61] Li H. (2018). Minimap2: Pairwise alignment for nucleotide sequences. Bioinformatics.

[62] Kovaka S., Zimin A.V., Pertea G.M., Razaghi R., Salzberg S.L., Pertea M. (2019). Transcriptome assembly from long-read RNA-seq alignments with StringTie2. Genome Biol..

[63] Robinson J.T., Thorvaldsdóttir H., Winckler W., Guttman M., Lander E.S., Getz G., Mesirov J.P. (2011). Integrative genomics viewer. Nat. Biotechnol..

[64] Love M.I., Huber W., Anders S. (2014). Moderated estimation of fold change and dispersion for RNA-seq data with DESeq2. Genome Biol..

[65] Xie C., Mao X., Huang J., Ding Y., Wu J., Dong S., Kong L., Gao G., Li C.-Y., Wei L. (2011). KOBAS 2.0: A web server for annotation and identification of enriched pathways and diseases. Nucleic Acids Res..

[66] Schmittgen T.D., Livak K.J. (2008). Analyzing real-time PCR data by the comparative CT method. Nat. Protoc..

