# Supplementary material for: Transcriptomics Analysis Reveals a Putative Role for Hormone Signaling and MADS-Box Genes in Mature Chestnut Shoots Rooting Recalcitrance
Source: Plants (Basel). 2022 Dec 13;11(24):3486. doi: 10.3390/plants11243486 (PMC9786281; doi:10.3390/plants11243486)
Supplement: Supplementary file 1 [file plants-11-03486-s001.zip › Supplemental Figure S1.pptx]

## Slide 1
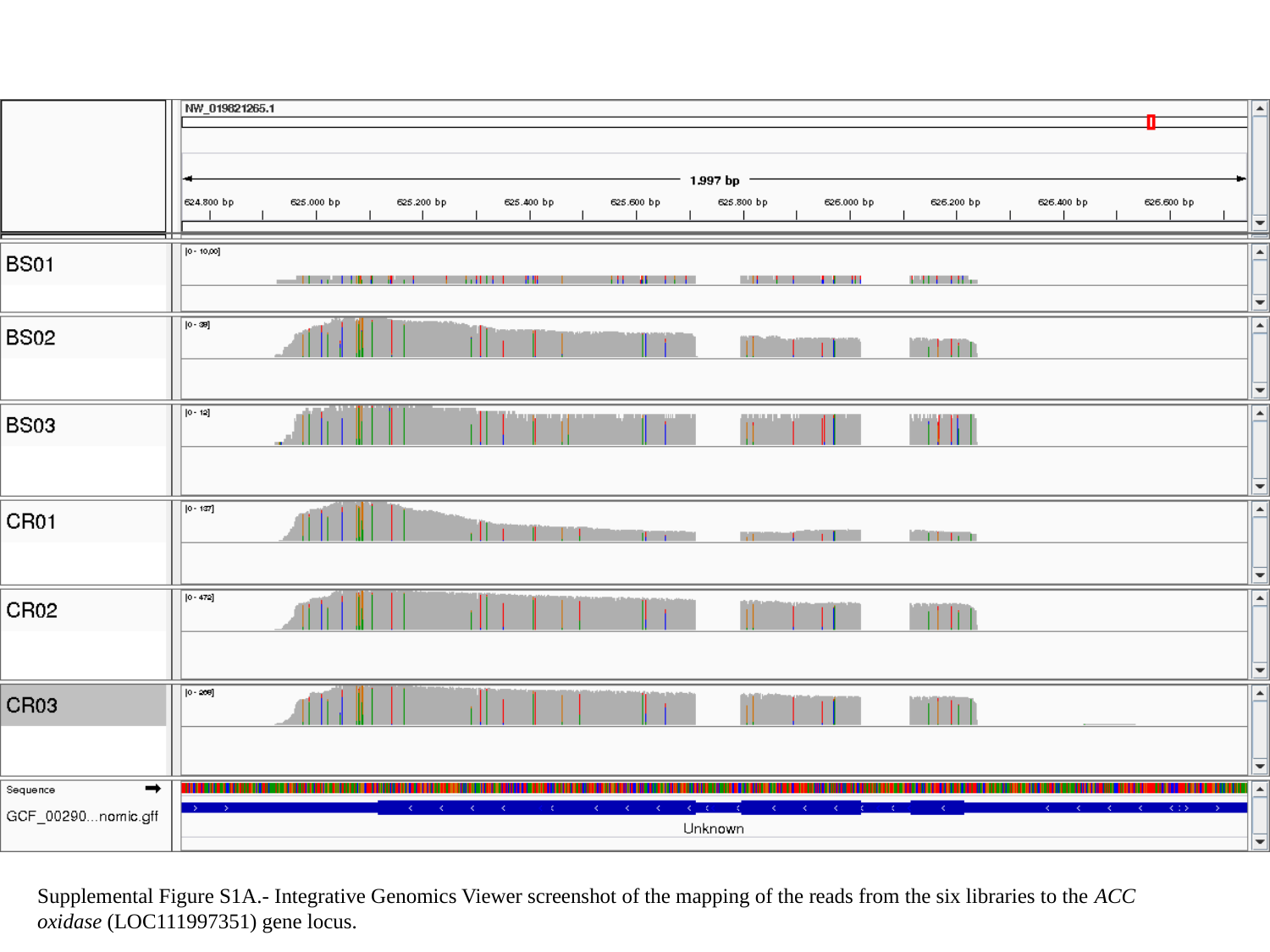

Supplemental Figure S1A.- Integrative Genomics Viewer screenshot of the mapping of the reads from the six libraries to the ACC oxidase (LOC111997351) gene locus.

## Slide 2
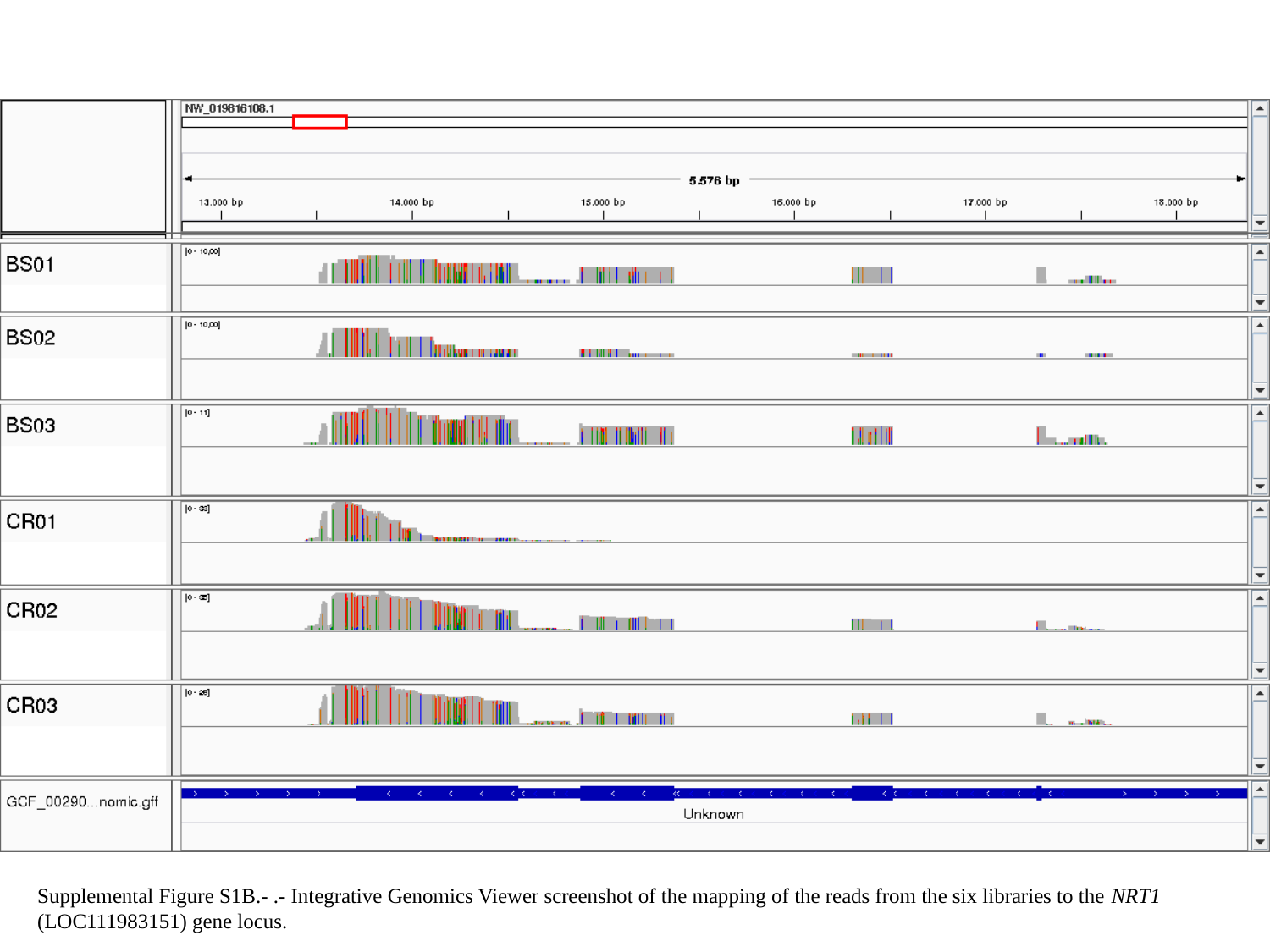

Supplemental Figure S1B.- .- Integrative Genomics Viewer screenshot of the mapping of the reads from the six libraries to the NRT1 (LOC111983151) gene locus.

## Slide 3
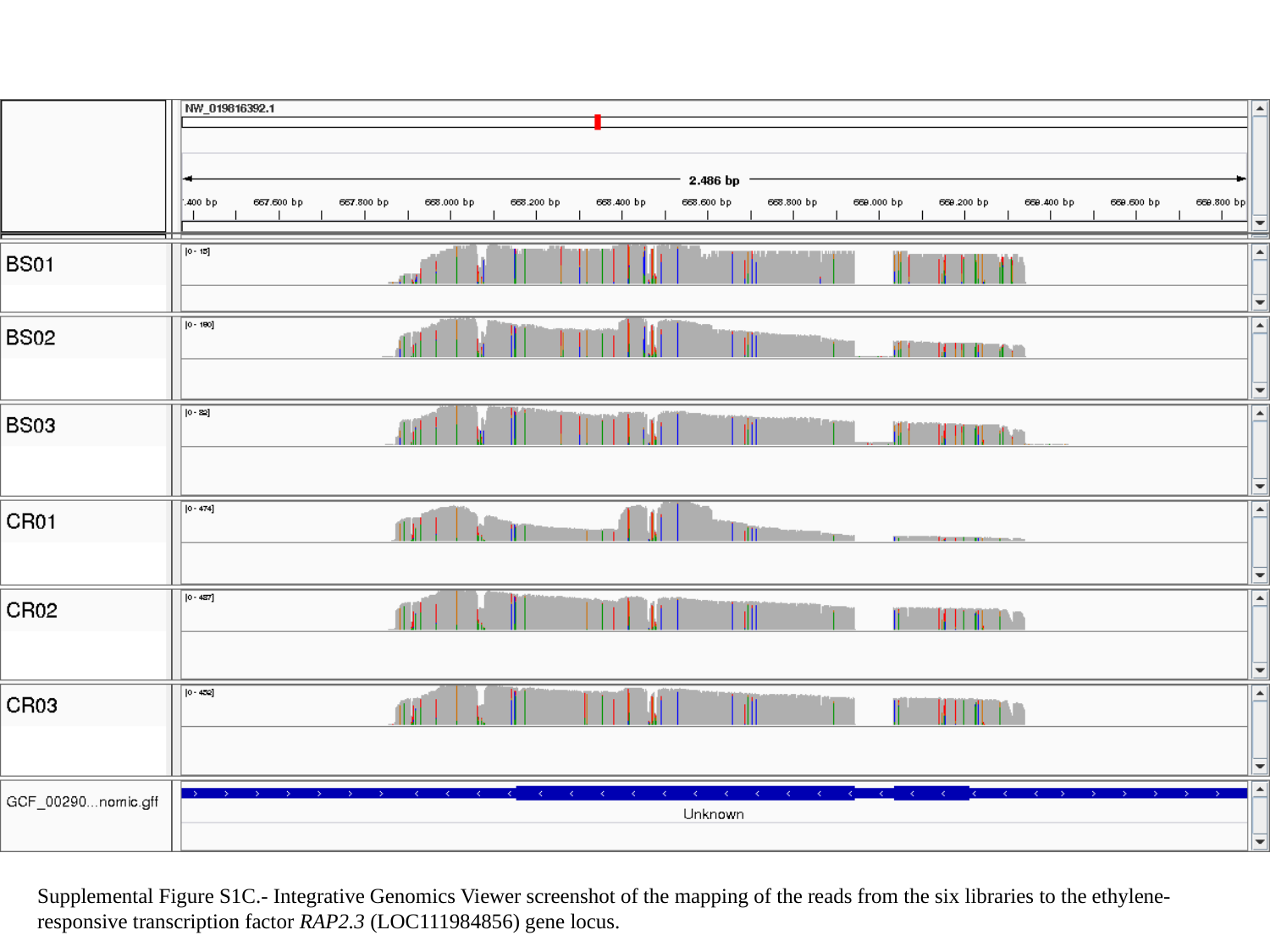

Supplemental Figure S1C.- Integrative Genomics Viewer screenshot of the mapping of the reads from the six libraries to the ethylene-responsive transcription factor RAP2.3 (LOC111984856) gene locus.

## Slide 4
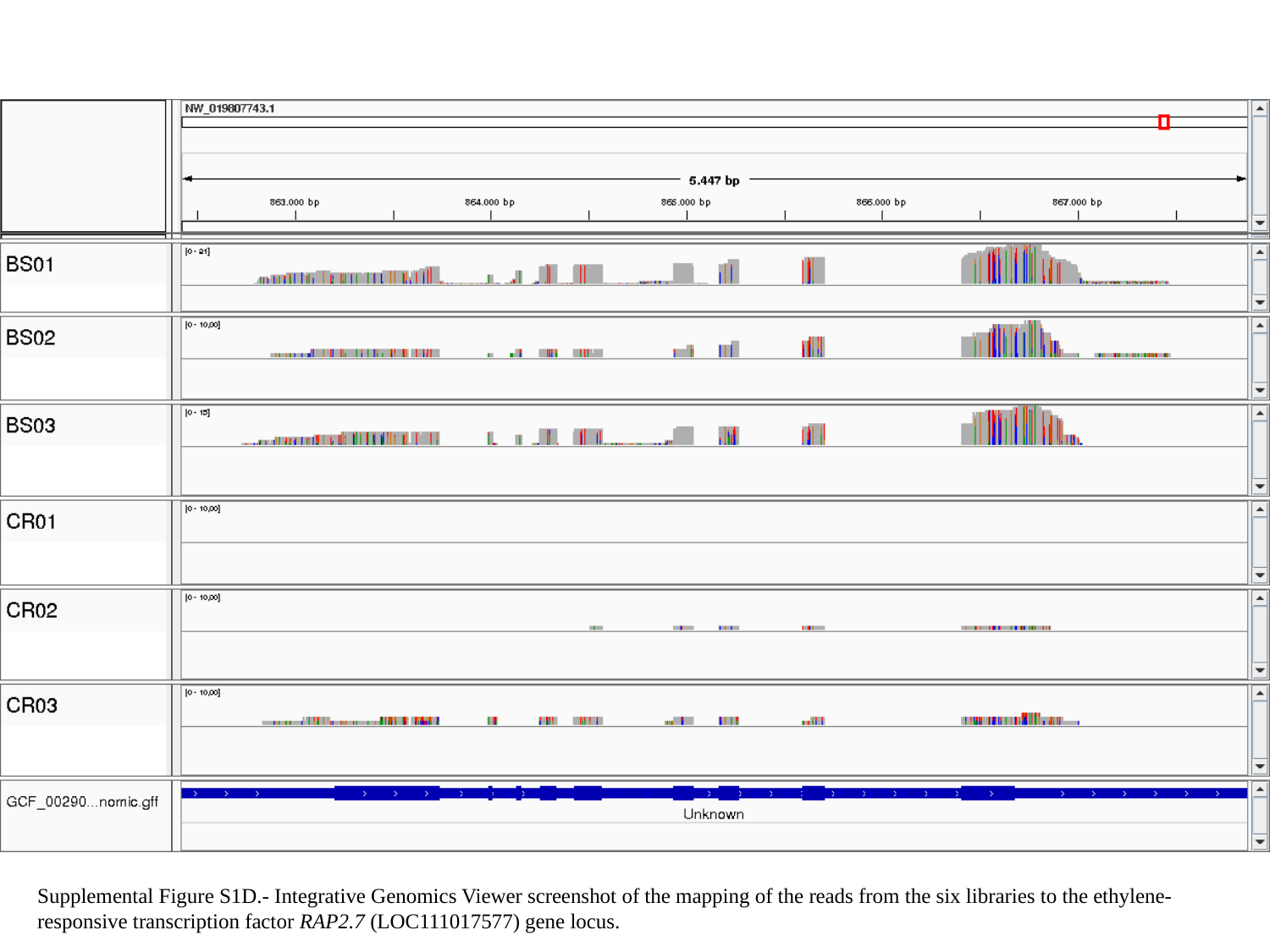

Supplemental Figure S1D.- Integrative Genomics Viewer screenshot of the mapping of the reads from the six libraries to the ethylene-responsive transcription factor RAP2.7 (LOC111017577) gene locus.
