# Supplementary material for: Transcriptomics Analysis Reveals a Putative Role for Hormone Signaling and MADS-Box Genes in Mature Chestnut Shoots Rooting Recalcitrance
Source: Plants (Basel). 2022 Dec 13;11(24):3486. doi: 10.3390/plants11243486 (PMC9786281; doi:10.3390/plants11243486)
Supplement: Supplementary file 1 [file plants-11-03486-s001.zip › Supplemental Figure S4.pptx]

## Slide 1
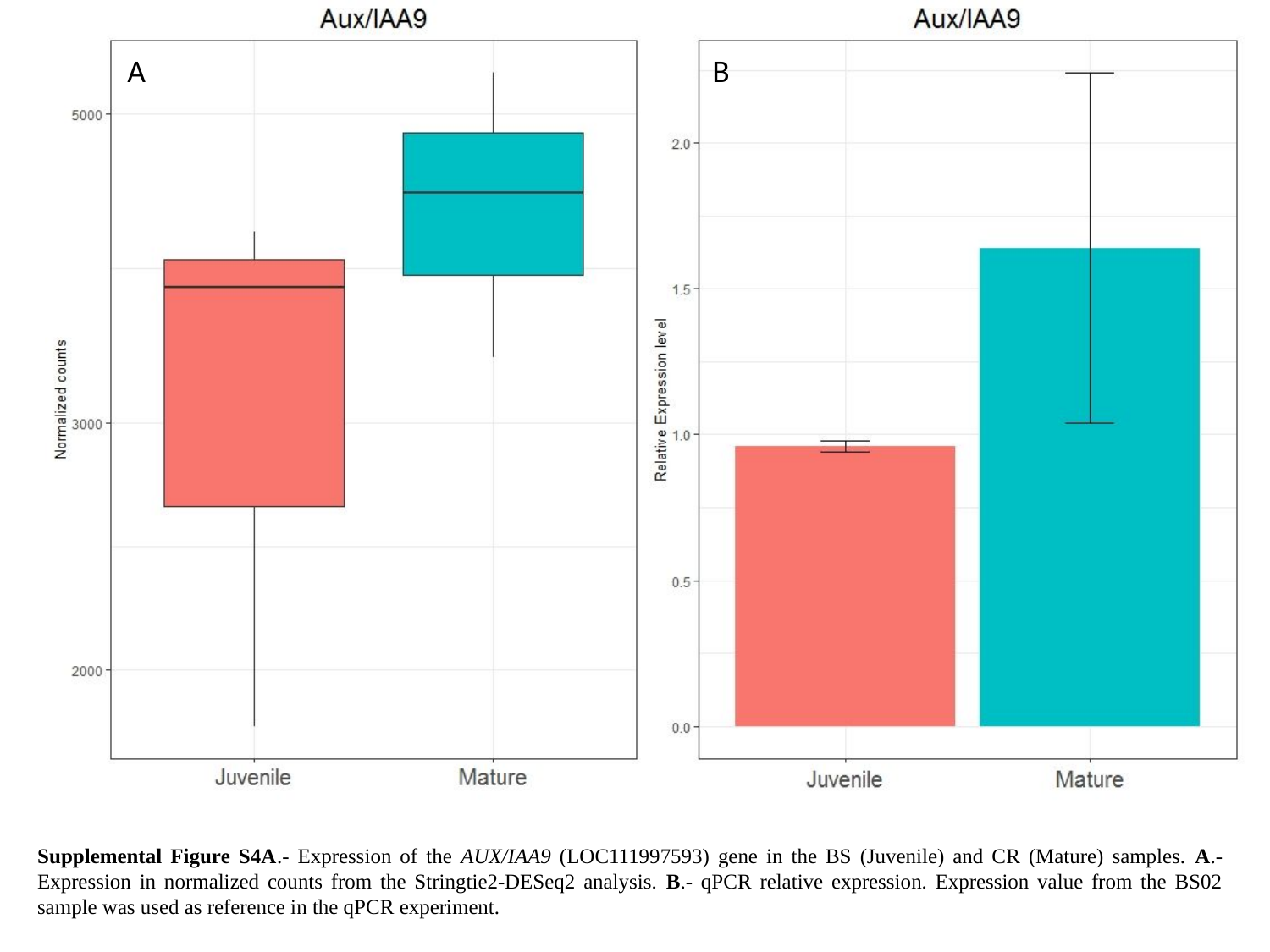

A
B
Supplemental Figure S4A.- Expression of the AUX/IAA9 (LOC111997593) gene in the BS (Juvenile) and CR (Mature) samples. A.- Expression in normalized counts from the Stringtie2-DESeq2 analysis. B.- qPCR relative expression. Expression value from the BS02 sample was used as reference in the qPCR experiment.

## Slide 2
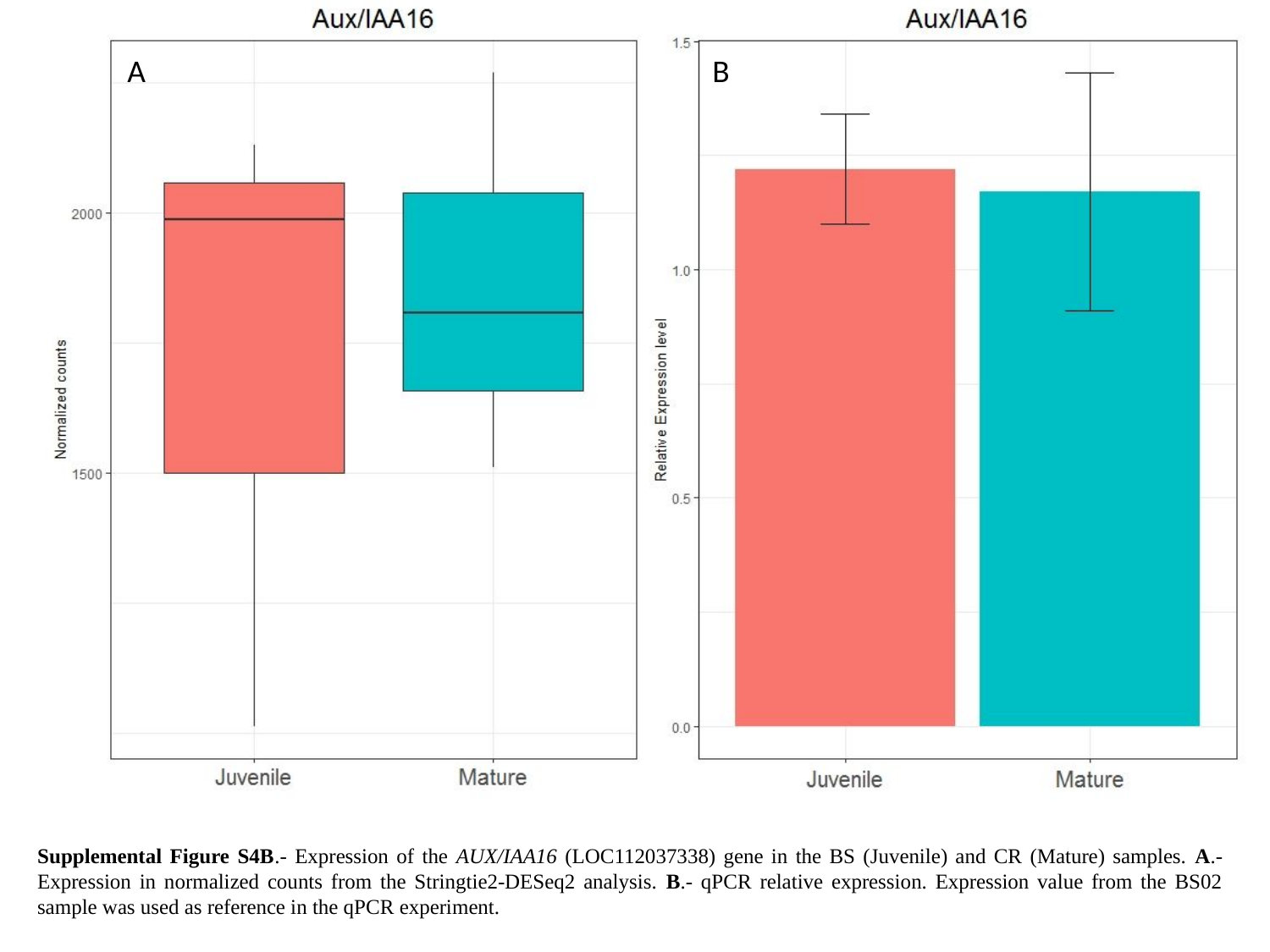

A
B
Supplemental Figure S4B.- Expression of the AUX/IAA16 (LOC112037338) gene in the BS (Juvenile) and CR (Mature) samples. A.- Expression in normalized counts from the Stringtie2-DESeq2 analysis. B.- qPCR relative expression. Expression value from the BS02 sample was used as reference in the qPCR experiment.

## Slide 3
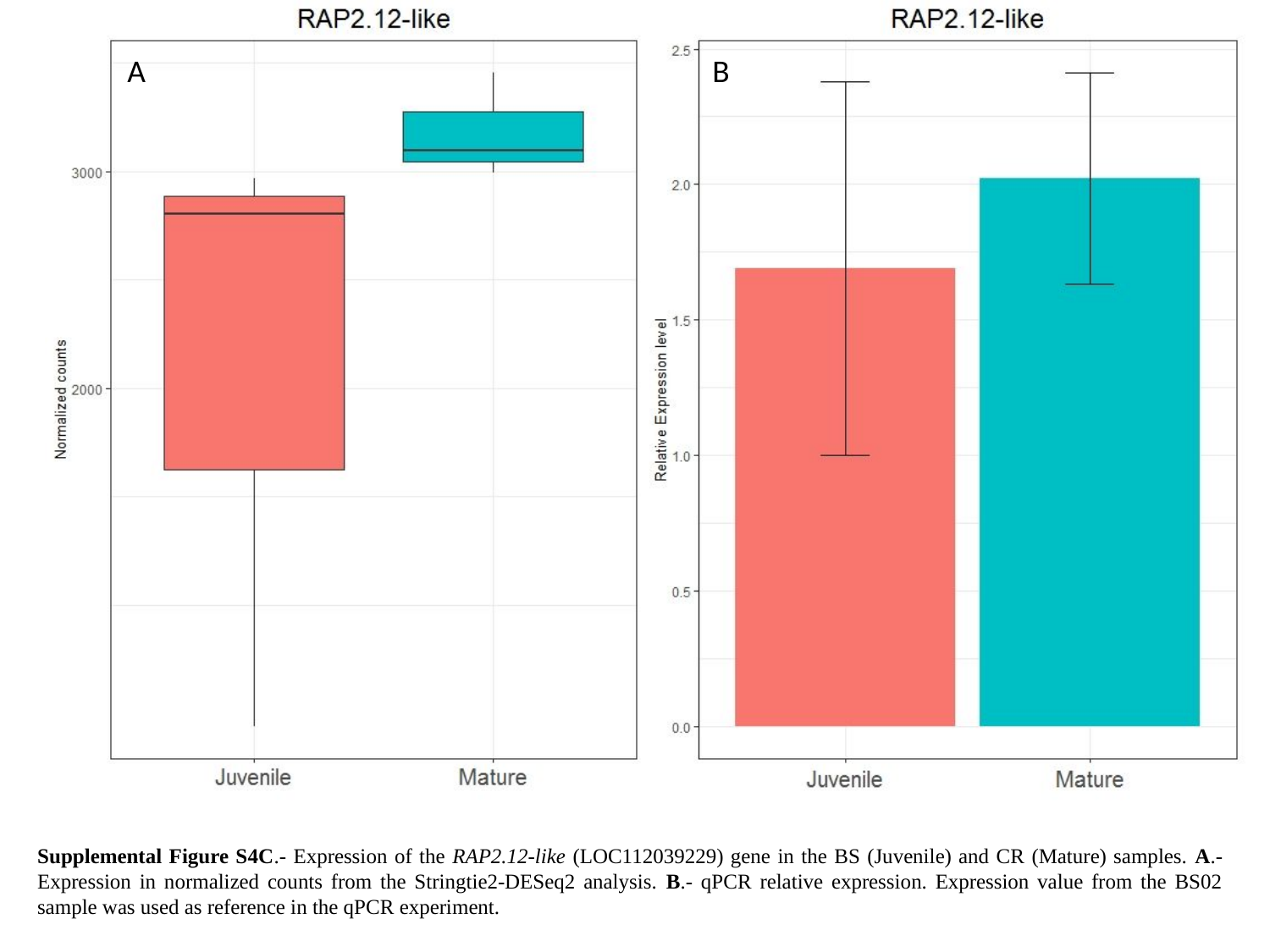

A
B
Supplemental Figure S4C.- Expression of the RAP2.12-like (LOC112039229) gene in the BS (Juvenile) and CR (Mature) samples. A.- Expression in normalized counts from the Stringtie2-DESeq2 analysis. B.- qPCR relative expression. Expression value from the BS02 sample was used as reference in the qPCR experiment.

## Slide 4
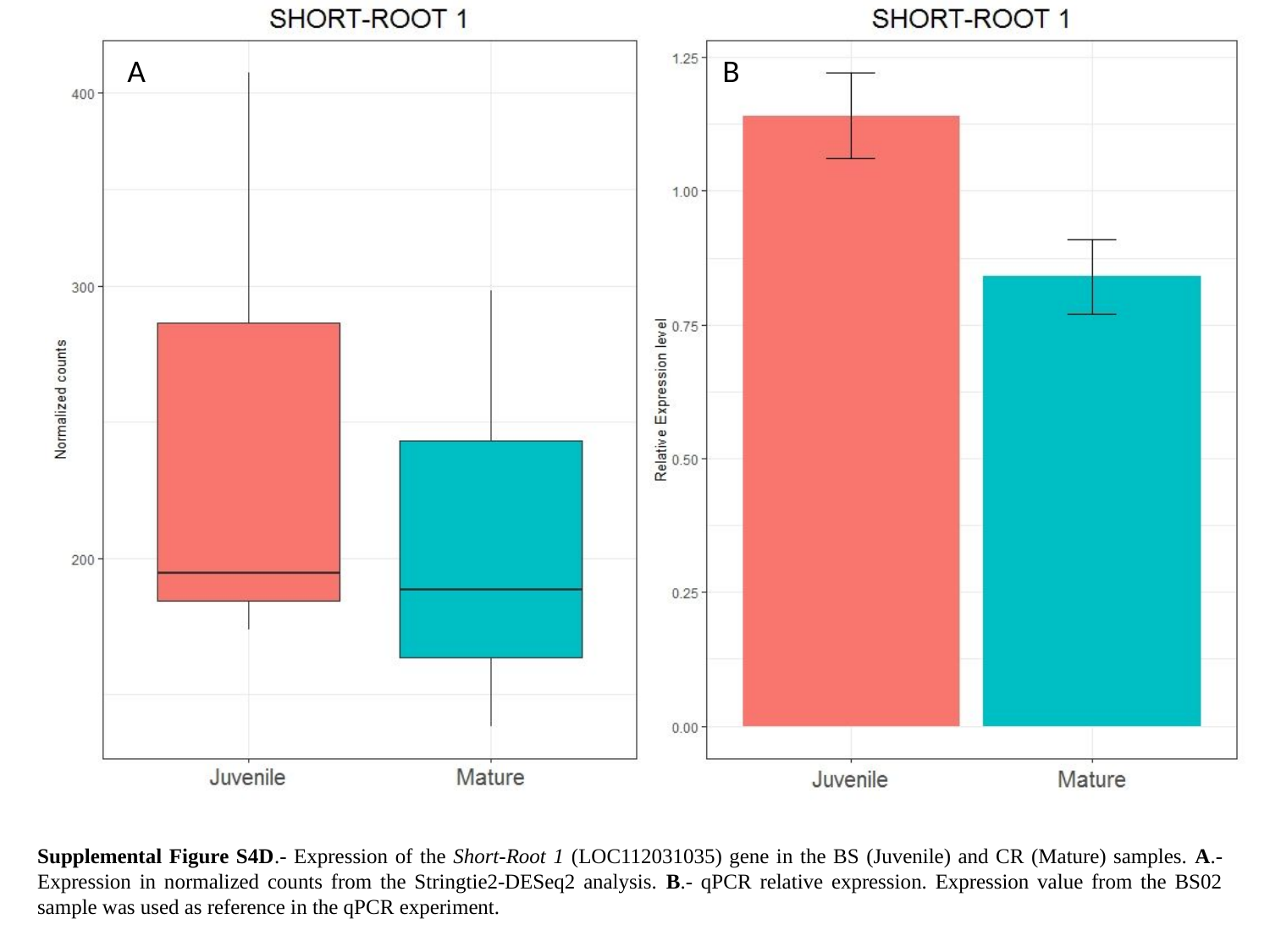

A
B
Supplemental Figure S4D.- Expression of the Short-Root 1 (LOC112031035) gene in the BS (Juvenile) and CR (Mature) samples. A.- Expression in normalized counts from the Stringtie2-DESeq2 analysis. B.- qPCR relative expression. Expression value from the BS02 sample was used as reference in the qPCR experiment.

## Slide 5
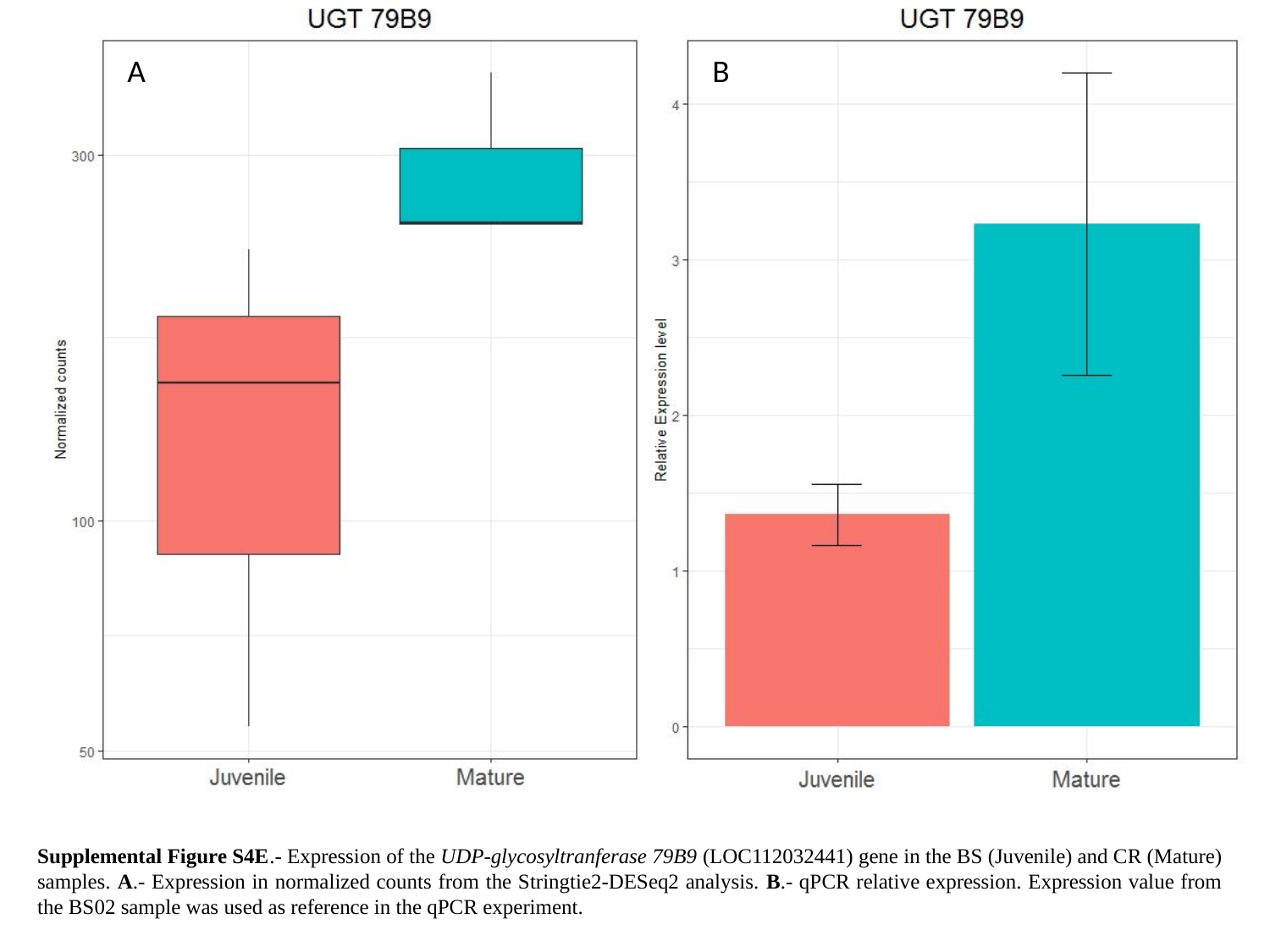

A
B
Supplemental Figure S4E.- Expression of the UDP-glycosyltranferase 79B9 (LOC112032441) gene in the BS (Juvenile) and CR (Mature) samples. A.- Expression in normalized counts from the Stringtie2-DESeq2 analysis. B.- qPCR relative expression. Expression value from the BS02 sample was used as reference in the qPCR experiment.

## Slide 6
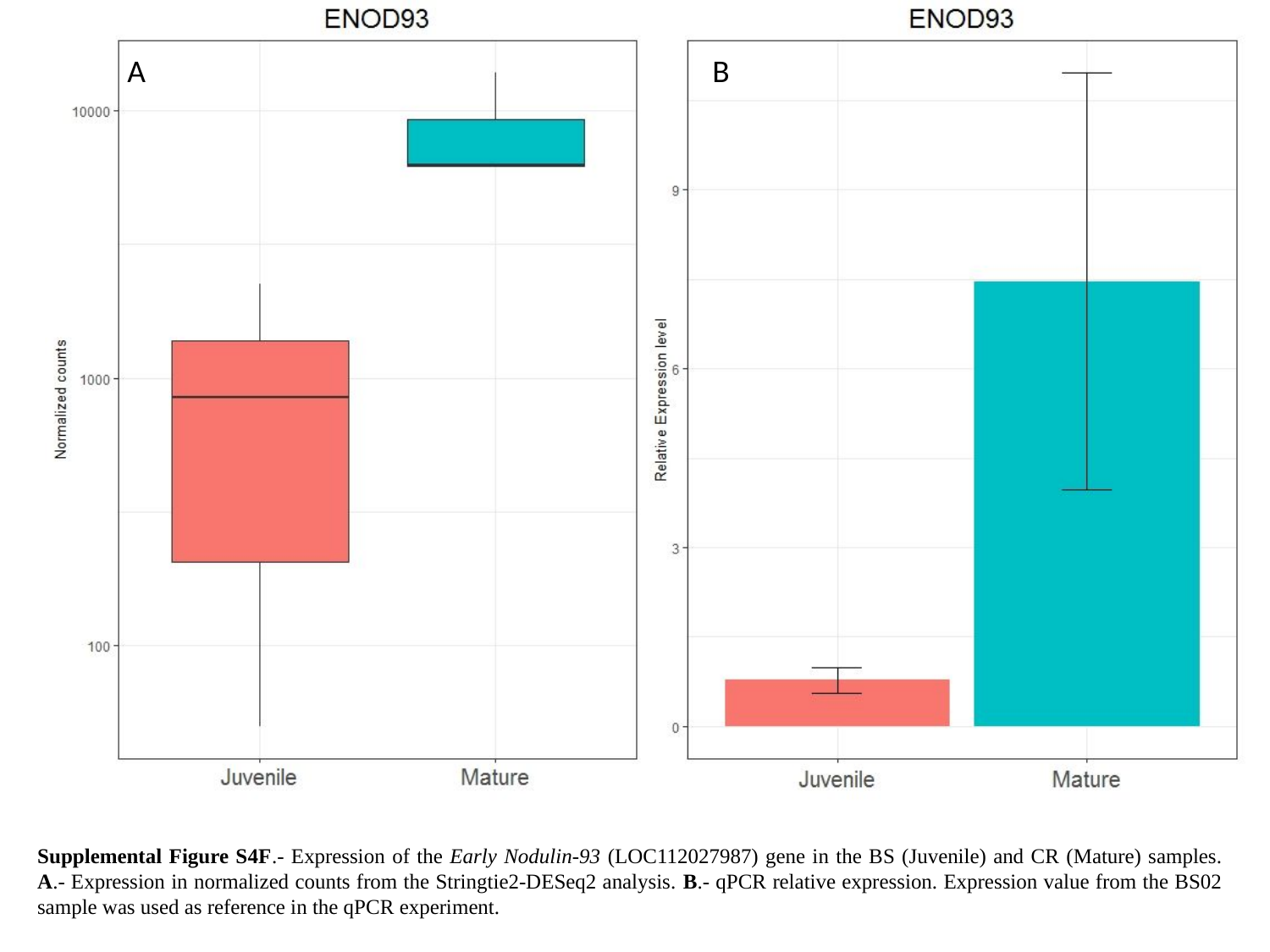

A
B
Supplemental Figure S4F.- Expression of the Early Nodulin-93 (LOC112027987) gene in the BS (Juvenile) and CR (Mature) samples. A.- Expression in normalized counts from the Stringtie2-DESeq2 analysis. B.- qPCR relative expression. Expression value from the BS02 sample was used as reference in the qPCR experiment.
