# Supplementary figures and images for: Transcriptomics Analysis Reveals a Putative Role for Hormone Signaling and MADS-Box Genes in Mature Chestnut Shoots Rooting Recalcitrance
Source: Plants (Basel). 2022 Dec 13;11(24):3486. doi: 10.3390/plants11243486 (PMC9786281; doi:10.3390/plants11243486)

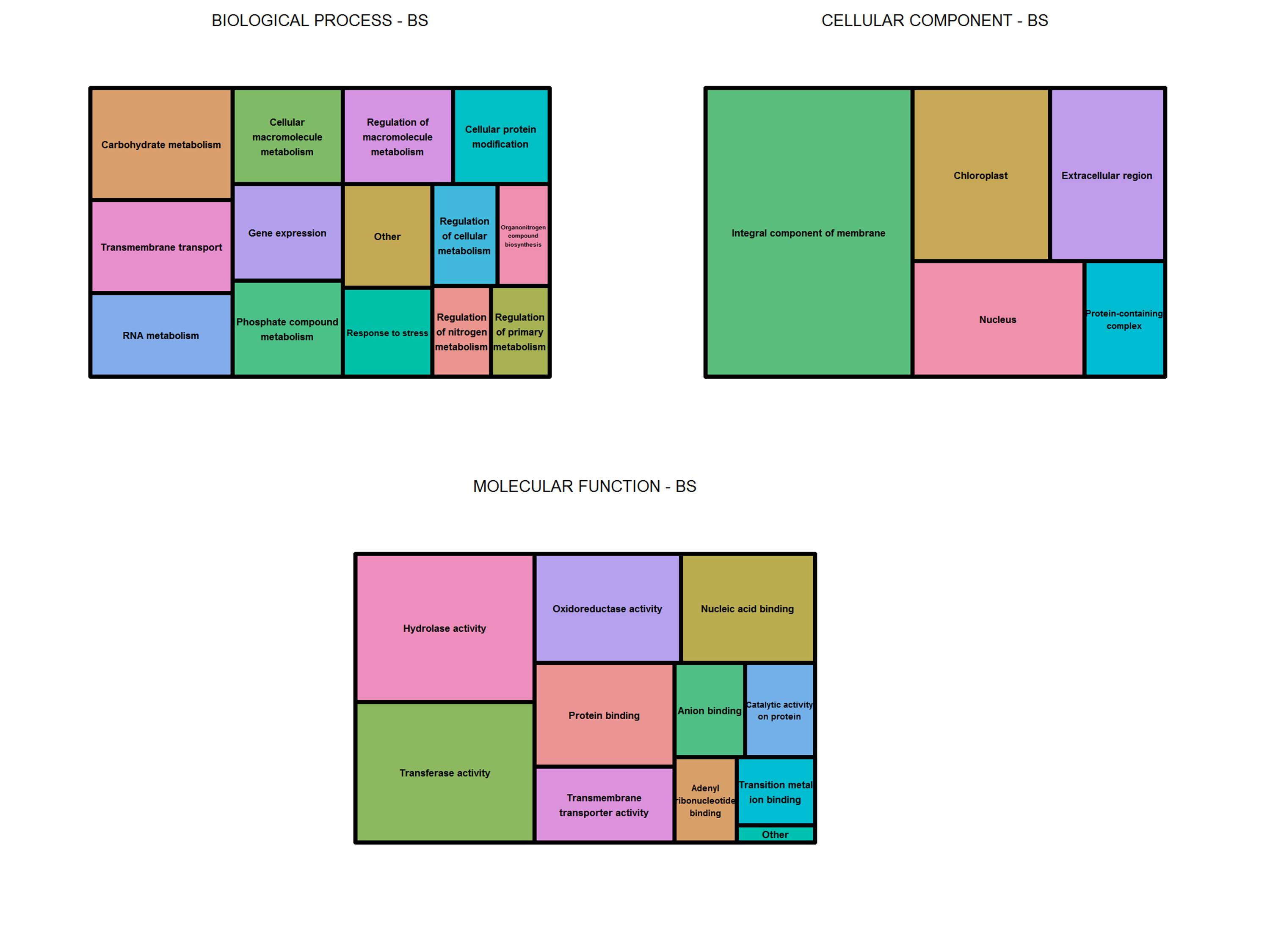

Supplement: Supplementary file 1 [file plants-11-03486-s001.zip › Supplemental Figure S2.jpg]

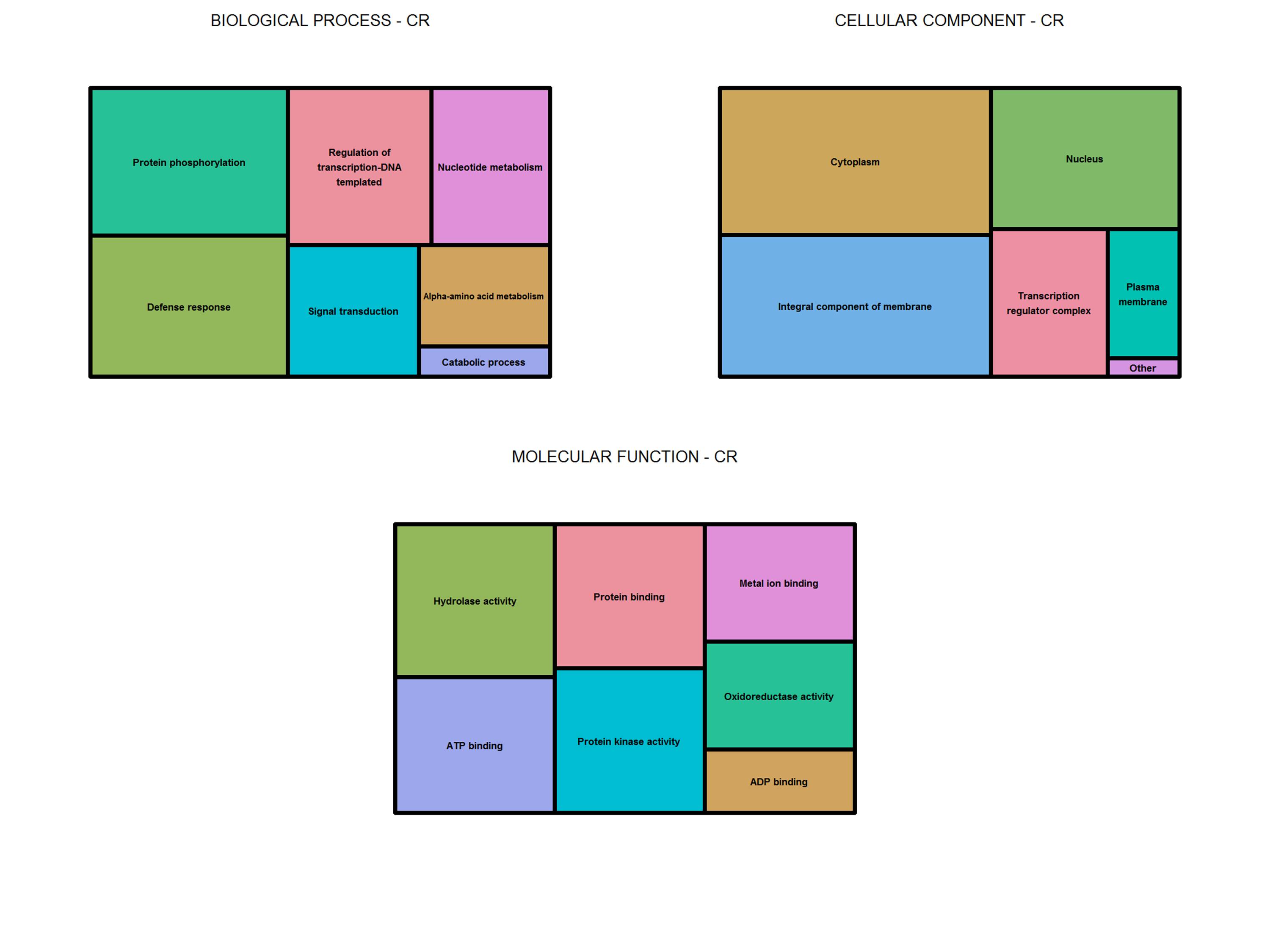

Supplement: Supplementary file 1 [file plants-11-03486-s001.zip › Supplemental Figure S3.jpg]
